# Individually Prescribed In-Bed Exergaming Using Nintendo Ring Fit Adventure in Posttransplant Inpatients With Marked Functional Impairment After Intensive Care: Prospective Single-Arm Feasibility Study

**DOI:** 10.2196/95037

**Published:** 2026-10-20

**Authors:** Yong Hoe Koo, Woo Chul Son, In Ho Woo, Ye Ji Kim, Soo Hoon Yoon, Ji Hye Kim, Young Cheon Song, Ho Cheol Kim, Dong-Hwan Jung, Jiwon Ryu, Gwanseob Shin, Jaesoon Choi, Won Kim

**Affiliations:** 1 Department of Rehabilitation Medicine, Asan Medical Center, University of Ulsan College of Medicine, 88, Olympic-ro 43-gil, Songpa-gu, Seoul, 05505, Republic of Korea, 82 -2-3010-1694, 82 -2-3010-6964; 2 Asan Institute for Life Sciences, Asan Medical Center, University of Ulsan College of Medicine, Seoul, Republic of Korea; 3 Department of Nursing, Asan Medical Center, Seoul, Republic of Korea; 4 Department of Pulmonary and Critical Care Medicine, Asan Medical Center, University of Ulsan College of Medicine, Seoul, Republic of Korea; 5 Department of Surgery, Asan Medical Center, University of Ulsan College of Medicine, Seoul, Republic of Korea; 6 Department of Biomedical Engineering, Ulsan National Institute of Science and Technology, Ulsan, Republic of Korea; 7 Department of Biomedical Engineering, Asan Medical Center, University of Ulsan College of Medicine, Seoul, Republic of Korea; 8 Biomedical Engineering Research Center, Asan Institute for Life Sciences, Asan Medical Center, Seoul, Republic of Korea

**Keywords:** exergaming, rehabilitation, critical illness, intensive care unit-acquired weakness, organ transplantation

## Abstract

**Background:**

Early rehabilitation is important after transplantation and critical illness, but profound weakness, fatigue, and motivational, psychological, and safety barriers may limit participation. Exergaming may facilitate rehabilitation, but the feasibility of full-body in-bed exergaming in this population remains unclear.

**Objective:**

We evaluated the feasibility and observed safety of individually prescribed in-bed exergaming using Nintendo Ring Fit Adventure (NFA; Nintendo Co, Ltd) in posttransplant inpatients after intensive care unit (ICU) care.

**Methods:**

This prospective, single-center, single-arm interventional feasibility study (October 2024-October 2025) enrolled 26 posttransplant adults with prior ICU care and Functional Ambulation Category (FAC) scores ≤3. Participants were referred after ward transfer by transplant-team advanced practice nurses, and the principal investigator confirmed eligibility based on prespecified inclusion and exclusion criteria. Ten individually prescribed, supervised in-bed NFA sessions, each scheduled for 30 minutes, including setup and operation, were planned alongside usual physical therapy. Data were obtained from medical records, standardized session logs, and prespecified clinical and questionnaire assessments. Outcomes included retention; session delivery, completion, and interruptions; exercise-item completion and interruptions; session-terminating adverse events; net exercise time; satisfaction; and exploratory strength, mobility, and health-related quality-of-life changes.

**Results:**

Participants (mean age 53.0, SD 12.4 y; 14 men) included 23 liver and 3 bilateral lung transplant recipients. Mean ICU stay and mechanical ventilation duration were 24.3 (SD 11.0) days and 21.3 (SD 11.6) days, respectively; median baseline FAC and Medical Research Council Sum Score were 0.5 (IQR 0.0-1.0) and 38.0 (IQR 30.5-42.0), respectively. None had an FAC score above 2. Twenty completed the final assessment (20/26, 76.9%, 95% CI 57.9%-89%). Of 227 delivered sessions, 223 (98.2%, 95% CI 95.8%-100%) were completed. Of 2161 prescribed exercise items, 2072 (95.9%, 95% CI 93.4%-97.9%) were completed. No session-terminating adverse events were observed (0/227, 0%, 95% CI 0%-1.6%). Median within-participant net exercise time increased by 159.0 (IQR 35.5-296.3) seconds from sessions 1 to 10 (95% CI 81.0-284.0; Wilcoxon signed-rank test, *P*<.001). Friedman tests followed by Bonferroni-adjusted Wilcoxon signed-rank tests (2-sided α=.05) showed baseline-to-final median within-participant changes of 8.0 Medical Research Council Sum Score points (IQR 2.3-10.3; 95% CI 4.0-10.0, *P*=.004), 2.0 Intensive Care Unit Mobility Scale points (IQR 1.0-4.3; 95% CI 1.0-3.5, *P*<.001), and 15.0 EQ-VAS points (IQR 0.0-30.0; 95% CI 5.0-25.0, *P*=.009). Questionnaire responses were favorable.

**Conclusions:**

Under therapist supervision, the individually prescribed in-bed NFA program showed favorable operational feasibility, with no session-terminating adverse events. By combining the accessibility of a commercial off-the-shelf exergame with individualized exercise prescription, the program extended bedside exergaming to posttransplant inpatients with substantial functional impairment after ICU care who had not achieved independent ambulation.

## Introduction

Advances in critical-care medicine have substantially improved survival after severe illness and complex surgery, including organ transplantation. However, following critical illness or organ transplantation, a substantial proportion of individuals experience functional decline characterized by sarcopenia and intensive care unit (ICU)-related neuromuscular impairment, including intensive care unit–acquired weakness (ICU-AW) [1-3]. These impairments often persist beyond the acute phase, contributing to prolonged disability and reduced health-related quality of life (HR-QOL) [2,4-6]. Early rehabilitation and mobilization are therefore key strategies for mitigating muscle wasting and promoting functional recovery [3,5,7,8], particularly in transplant recipients, in whom postoperative catabolic and nutritional disturbances and the metabolic effects of immunosuppressive therapy may create additional barriers to physical recovery [3,9].

Despite the growing emphasis on early mobilization, delivering sufficient rehabilitation to patients with critical illness-related functional impairment remains challenging. For high-acuity patients who have not achieved independent ambulation, profound weakness, fatigue, and psychological barriers can limit sustained exercise participation [1,10-12]. These barriers may be especially relevant to posttransplant patients, in whom transplant-related fatigue, fear of movement, and low self-efficacy can further limit exercise participation despite substantial rehabilitation needs [13-15]. Systemic constraints, including limited staffing and reimbursement structures, may also restrict the frequency and duration of supervised one-to-one therapy sessions [16-18]. Together, these patient- and system-level barriers contribute to prolonged inactivity during hospitalization.

In this context, technology-assisted rehabilitation has been explored as an adjunct to conventional therapist-led care. Exergaming integrates physical exercise with interactive video game elements, often using motion-sensing technology [12,19-21]. A distinguishing feature of exergaming is that it embeds physical movements within goal-directed, game-like tasks and provides immediate performance feedback [20,22-25], features that may support enjoyment, motivation, and engagement in ways consistent with self-determination theory [22,26]. Exergaming-based interventions have demonstrated feasibility and acceptability across diverse populations [19,27-29]. Adapted interactive virtual reality has also been feasible for supine upper-limb exercise in bed-restricted patients with prolonged critical illness [30]. Nevertheless, evidence regarding the feasibility and safety of exergaming during or after critical illness remains limited [25,30-32]. To our knowledge, in-bed full-body exergaming incorporating upper and lower extremity and trunk movements has not been studied in posttransplant recipients with post-ICU functional decline and profound mobility impairment.

Nintendo Ring Fit Adventure (NFA) is a commercially available video game that enables full-body exercise through motion-sensing technology. Although not developed for clinical use, NFA’s custom mode allows built-in exercise items to be selected and sequenced according to clinical status and tolerance, while some exercise items (Fit Skills) have movement patterns amenable to modification for supported in-bed performance. Together, these features may facilitate bedside application in patients with marked functional impairment and limited physiological reserve. Published studies on NFA have mainly been conducted in populations with relatively preserved physical function [33-35], whereas evidence for its use in medically complex inpatients with severe functional impairment after ICU care or transplantation remains lacking.

Therefore, we evaluated the feasibility and observed safety of NFA as an individually prescribed in-bed exergaming rehabilitation program for posttransplant inpatients with marked functional impairment after ICU care. Secondary objectives were to (1) describe the characteristics and longitudinal trends in exercise performance and (2) descriptively characterize physical function and HR-QOL over the intervention period and assess patient satisfaction.

## Methods

### Conditions and Design

This prospective, single-center, single-arm interventional feasibility study was conducted at Asan Medical Center (a tertiary university hospital in Seoul, Republic of Korea) between October 2024 and October 2025. The trial was retrospectively registered at ClinicalTrials.gov on April 20, 2025 (NCT06937463). Reporting of this study was guided by the Journal Article Reporting Standards for Quantitative Research [36], and the intervention was described according to the TIDieR (Template for Intervention Description and Replication) framework [37] (Checklist 1).

### Inclusion, Exclusion, and Sampling Procedures

Patients were included if they were organ transplant recipients, aged 18 years or older, had received ICU care during the hospitalization in which transplantation was performed, and had not achieved independent ambulation; inability to ambulate independently was defined as a Functional Ambulation Category (FAC) score of 3 or less. Patients were excluded if they met any of the following criteria: (1) inability to follow instructions owing to cognitive impairment or sedation; (2) hemodynamic instability precluding rehabilitation (uncontrolled shock with persistent hypotension despite vasopressor therapy, an arrhythmia causing hemodynamic compromise, or uncontrolled active bleeding); (3) severe musculoskeletal limitations (eg, unstable fractures or recent amputations) preventing exercise participation; and (4) significant sensory impairments (eg, hearing loss or visual impairments) hindering interaction with the exergaming system. A convenience sampling approach was used. After transfer from the ICU to a general ward, potentially eligible patients were referred by advanced practice nurses from the relevant transplant teams to the principal investigator for study participation. The principal investigator, a physiatrist, subsequently confirmed eligibility, obtained written informed consent, and finalized enrollment. The intervention was delivered exclusively at the bedside on the general ward.

### Participant Characteristics

Baseline characteristics specified for descriptive reporting included demographic and anthropometric characteristics, cognitive status, vital signs, transplantation- and hospitalization-related characteristics, ICU and mechanical ventilation courses, respiratory support, timing of intervention initiation, and baseline muscle strength and mobility.

### Intervention Protocol and Instrumentation

#### NFA Hardware and Software

The NFA system comprises a Nintendo Switch console, a ring-shaped resistance controller (Ring-Con), and a leg-mounted motion sensor (leg strap), which together capture upper-extremity, lower-extremity, and trunk movements. The Ring-Con detects pressing and pulling force and, together with the attached Joy-Con, captures arm movement, enabling real-time interaction with the game. The software links these motion inputs to interactive exercise tasks. For this study, NFA custom mode was used to individualize exercise prescription by selecting and sequencing up to 10 of 43 built-in Fit Skills exercises for each session (Figure 1A).

**Figure 1.**
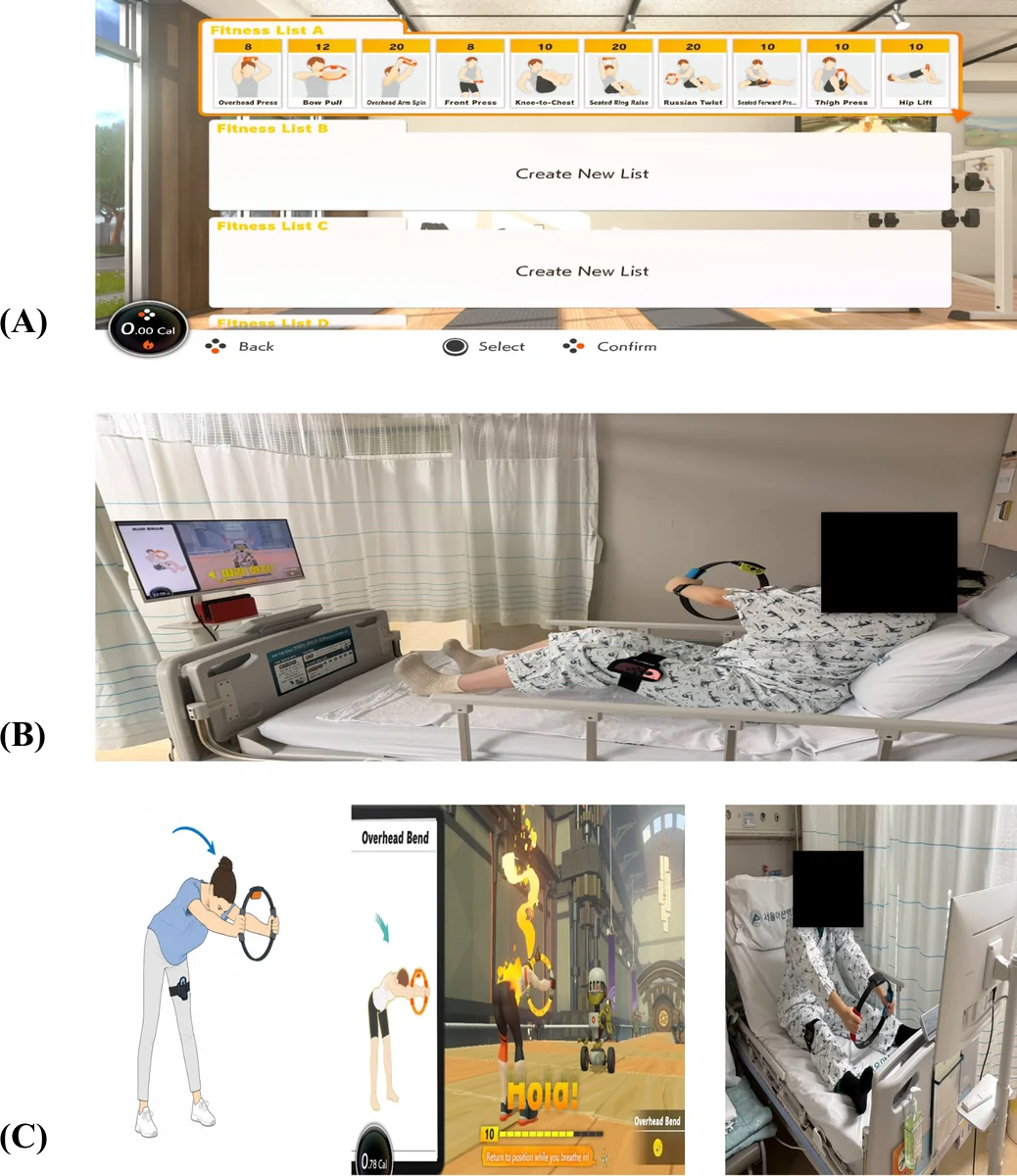
Implementation of the in-bed exergaming program for posttransplant inpatients after intensive care. (A) Custom mode interface of Nintendo Ring Fit Adventure (NFA), used to create individualized exercise item lists. (B) Representative bedside setup. The height-adjustable monitor was positioned at the foot of the bed within the participant’s line of sight, with the Nintendo Switch console placed immediately below the monitor. Depending on their functional status and the selected exercise item, participants performed the exercises in a supported seated or semirecumbent position. The Ring-Con, fitted with one Joy-Con controller, was held with both hands, while the second Joy-Con controller was secured to the thigh using the leg strap. (C) Comparison of standard and modified exercise implementations: (left) an illustration based on an NFA reference image depicting the standard exercise item posture; (middle) in-game screenshot of the corresponding movement; and (right) modified exercise posture adapted for in-bed performance. The adaptation retained the primary target body region but was not considered biomechanically equivalent to the standard exercise item.

#### NFA-Based In-Bed Exergaming Program

The program comprised 10 planned bedside sessions, scheduled once daily when the participant’s clinical condition and hospital schedule permitted. Before each session, a physician confirmed that the participant was clinically able to participate. A certified physical therapist then brought the NFA equipment and a height-adjustable monitor to the participant’s room and set them up at the bedside, with the console positioned below the monitor at the foot of the bed. This setup allowed the participant a clear view of the game screen while using the Ring-Con with the leg strap secured to the thigh in a supine, semirecumbent, or seated-in-bed position (Figure 1B). Each session was planned within a 30-minute window, including equipment setup and operation. Before each session, the same treating physical therapist calibrated the Ring-Con using the therapist’s own maximum force rather than the participant’s force to standardize the setting across participants and sessions. Before the first session, the therapist provided a brief explanation and demonstration of device use and basic game-controlled movements.

For this study, 13 exercise items were selected by the physiatrists and physical therapists involved in the study a priori based on clinical judgment regarding their feasibility for modified in-bed performance and clinical relevance, while aiming to include exercises targeting the upper extremities, lower extremities, and trunk. Each exercise item was categorized according to the primary body region targeted. Exercise items originally designed for standing were adapted for in-bed use and performed in a seated position, with ranges of joint motion adjusted as needed while preserving the intended movement pattern (Figure 1C; more details in Multimedia Appendix 1).

All patients received an individually prescribed in-bed exergaming program in addition to usual physical therapy (PT). At the beginning of each session, a physical therapist assessed the clinical status (including general condition, strength, and range of motion) of the patient and selected up to 10 exercise items from the 13-item library, tailoring the combination of upper extremity, trunk, and lower extremity exercises to the individual. For each selected item, the therapist typically prescribed one set of 8‐14 repetitions. The number of repetitions was individualized based on an overall assessment of muscle strength, available range of motion, hemodynamic stability, and the participant’s response to exercise, including perceived exertion, pain, and fatigue. Following therapist demonstration, the participant briefly tested each prescribed item, and the therapist determined whether to include it in that day’s exercise item set. No prespecified quantitative intensity target was used, and the Fit Skills used in this study did not provide a separate difficulty setting within custom mode. Instead, exercise dose was individualized within and across sessions by adjusting the selected exercise items, the number of items, and the prescribed number of repetitions. As the participant’s clinical status and exercise tolerance improved, the therapist could modify the selected items and increase the number of items or prescribed repetitions. Whenever the patient could perform the movement through a substantial portion of the intended range of motion but could not achieve the full range, the therapist provided mild manual assistance as needed to facilitate movement toward the desired range while maintaining active participation.

#### Safety Monitoring

Clinical safety was monitored throughout each NFA session by the supervising physical therapist, who made the initial decision to continue, modify, or discontinue exercise. Heart rate (HR) and oxygen saturation were monitored during every intervention session for real-time application of the prespecified safety criteria. For analysis, only resting and peak HR values were formally recorded during sessions 1, 6, and 10. When additional clinical assessment was needed, the therapist conferred with the participant’s physician or advanced practice nurse. Prespecified safety criteria for pausing, modifying, or discontinuing exercise included respiratory distress (respiratory rate >35 breaths/min or use of accessory respiratory muscles), cyanosis, oxygen saturation below 90%, tachycardia (an increase in HR of ≥40 beats/min from baseline), hypotension (mean arterial pressure <60 mm Hg), participant intolerance (eg, diaphoresis, tremor, dizziness, or severe pain), unanticipated tube or line removal, and falls. If any of these criteria were met, exercise was paused or modified by reducing its intensity and could be resumed after recovery; the session was discontinued if the participant did not recover within 2 minutes. Immediate discontinuation occurred in the event of a fall or unplanned tube or line dislodgement. Sessions could also be interrupted in the absence of these criteria because of patient refusal or when the therapist determined that the participant was unable to continue for reasons such as fatigue or reduced tolerance.

#### Usual PT

Participants received usual PT according to standard institutional care. The therapy followed a stepwise ICU-AW mobilization protocol: stage 1, in-bed passive range-of-motion exercises; stage 2, in-bed active range-of-motion exercises; stage 3, edge-of-bed sitting; stage 4, standing; and stage 5, ambulation as tolerated with or without an assistive device. Progression was guided by the same safety criteria described in the “Safety Monitoring” subsection and stage-specific functional readiness, including the ability to follow commands, maintain head control, and generate sufficient upper- and lower-limb strength, as relevant to each successive stage. Usual PT was generally scheduled once daily, with each session lasting approximately 30 minutes; actual delivery varied according to the participant’s clinical condition and schedule.

### Measures and Covariates

#### Overview

Measures included participant-, session-, and exercise-item–level exercise metrics used to characterize operational feasibility, observed safety indicators, exploratory clinical outcomes, and patient-reported outcomes. No covariates were prespecified or included in adjusted analyses.

#### Exercise Metrics and Safety

At the session level, the metrics were interrupted sessions and net exercise time per session, defined as the cumulative duration of active exercise excluding the preparation time and rest periods. A session was considered interrupted if it was terminated before completion of the planned exercise set. At the exercise-item level, a prescribed item was defined as an exercise selected and configured in the NFA software at the beginning of the session. Prescribed items included in that day’s exercise set after brief testing were classified as attempted. Attempted items were considered completed when all prescribed repetitions were performed with correct form, as judged by the therapist, with or without manual assistance; items terminated before completing all prescribed repetitions were classified as interrupted. HR during exercise was monitored using an optical armband sensor (Verity Sense, Polar Electro). HR responses were assessed using resting HR before exercise, peak HR during exercise, and absolute HR change, calculated as peak HR minus resting HR. Any interruption due to a prespecified safety criterion was classified as a session-terminating adverse event. For all interruptions, the reason and the exercise item being performed were recorded.

#### Muscle Strength and Functional Measures

Overall muscle strength was assessed using the Medical Research Council Sum Score (MRC-SS), which grades six bilateral upper and lower limb muscle groups (shoulder abduction, elbow flexion, wrist extension, hip flexion, knee extension, and ankle dorsiflexion) on a 0‐5 scale (0: no visible contraction; 5: normal strength), yielding a composite score ranging from 0 to 60 [38]. Additional muscle strength measures included the handgrip strength (HGS) and knee extensor strength (KES), assessed using a digital hand dynamometer (TKK-5401, Takei Scientific Instruments) and a handheld dynamometer (Commander Echo MMT, JTECH Medical), respectively. HGS was measured with the participant in a seated or semi-Fowler position, with the dynamometer kept free from contact with the bed or body. KES was measured with the participant seated at the edge of the bed when possible or in a semi-Fowler position with knee flexion supported by a pillow. The dynamometer was positioned against the distal anterior tibia and manually stabilized by the therapist. Each side underwent 3 maximal-effort trials separated by 30-second rest intervals, and the highest of the 6 values was used for analysis. Mobility was assessed primarily using the Intensive Care Unit Mobility Scale (ICUMS) [39], which was designated as the principal exploratory mobility measure. The ICUMS is a 0‐10 ordinal scale that captures the highest level of mobility achieved, from passive movements in bed to independent ambulation. Additionally, as an ancillary measure, mobility was assessed using the FAC [40], a 6-point scale describing the level of assistance required for walking (0: nonfunctional ambulation, 1: continuous physical assistance, 2: intermittent physical assistance, 3: supervision or standby assistance, 4: independent on level surfaces, and 5: independent on all surfaces, including stairs). Functional lower-limb movement was evaluated using study-specific ordinal grading systems for the graded straight leg raise (SLR; grade 0: unable; grade 1: <30°; grade 2: ≥30° with <10 repetitions; grade 3: ≥30° with ≥10 repetitions) and bridge tests (grade 0: unable; grade 1: bridge height allowing a flat hand to pass; grade 2: bridge height allowing a fist to pass for <5 repetitions; and grade 3: the same height for ≥5 repetitions).

#### Patient-Reported Outcomes

Patient-reported outcomes included HR-QOL and acceptance of the NFA-based program. HR-QOL was assessed using the widely applied and validated EQ-5D-5L instrument [41]. The EQ-5D-5L descriptive system evaluates five domains (mobility, self-care, usual activities, pain or discomfort, and anxiety or depression), from which a summary index score is derived using the Korean population value set [42]. The instrument also includes the EQ-VAS reflecting the self-rated overall health status of the participant. Acceptance and user experience were evaluated using a study-specific self-reported satisfaction questionnaire composed of 11 items rated on a 5-point Likert scale. For descriptive presentation, the items were grouped by content into 3 groups: satisfaction and motivation, convenience and suitability, and perceived effectiveness of the program. The same questionnaire included a single item on perceived exercise intensity, rated on a 5-point difficulty scale (1: very easy, 3: appropriate, and 5: very difficult; Multimedia Appendix 2).

### Data Collection

A session log was completed by the physical therapist at every session to document the prescribed exercise items, their attempt and completion status, reasons for any session interruption or exercise-item interruption, and the net exercise time for that session. Standardized muscle strength, functional measures, and EQ-5D-5L scores were collected at sessions 1, 6, and 10, which corresponded to the baseline, midpoint, and final evaluations, respectively. The study-specific questionnaires were administered at the midpoint and final evaluations only. During sessions 1, 6, and 10, the HR was monitored continuously, and both the presession resting HR and in-session peak HR reached were recorded. Baseline demographic and clinical characteristics were obtained from electronic medical records.

### Quality of Measurements, Psychometrics, and Masking

Measurements were obtained by physical therapists using the standardized positioning, instructions, scoring procedures, and assessment schedules described in the preceding subsections of “Measures and Covariates.” Multiple maximal-effort trials were used for HGS and KES, and the MRC-SS, ICUMS, FAC, and EQ-5D-5L were administered according to their established scoring systems. Formal psychometric properties were unavailable for the study-specific graded SLR, bridge test, and satisfaction questionnaire. Masking was not applicable because all participants received the same intervention. Treating therapists also conducted outcome assessments and were not blinded to previous results. The questionnaires were distributed and nonanonymous but were completed independently without the therapist present.

### Sample Size, Power, and Precision

A pragmatic target of approximately 24 participants completing the final assessment was selected to support preliminary estimation of feasibility outcomes within the planned single-center recruitment period. Based on experience at our center, approximately 85% of enrolled participants were expected to complete the study; allowing for approximately 15% attrition, the enrollment target was set at 28 participants. At this sample size, an assumed completion proportion of 85% would yield a 2-sided 95% Wilson CI of approximately 68%‐94%. No formal hypothesis-testing power calculation was performed because this was a feasibility study. The predefined recruitment period ended before the target enrollment was reached.

### Data Diagnostics

Before analysis, the data were reviewed for completeness, out-of-range values, and internal inconsistencies. No participant was excluded after data collection solely on the basis of a statistical outlier, and no winsorization or data transformation was applied. Measurements that could not be obtained reliably were treated as unavailable, and missing values were not imputed. A Little test of missing completely at random was performed using repeated MRC-SS, ICUMS, EQ-VAS, and absolute HR change measures.

### Analytic Strategy

All statistical analyses were performed using SPSS Statistics (version 31.0; IBM Corp) and R software version 4.5.2 (R Foundation for Statistical Computing). Continuous variables were summarized as means (SDs) or medians (IQRs), as appropriate, and categorical variables as counts and percentages. At each assessment, clinical and patient-reported outcomes were summarized using all available observations. Paired changes were calculated among participants with data at both corresponding time points and summarized as median within-participant differences; 95% CIs were estimated using a nonparametric percentile bootstrap with 10,000 resamples. Changes in muscle strength, functional measures, HR-QOL, and absolute HR change across the 3 assessments were analyzed using Friedman tests in outcome-specific complete cases. Significant overall Friedman tests were followed by 2-sided Wilcoxon signed-rank tests, with *P* values Bonferroni-adjusted for the 3 comparisons within each outcome. Among final-evaluation completers, exploratory Spearman rank correlations were calculated between the number of usual PT sessions received during the intervention period and baseline-to-final changes in MRC-SS and ICUMS, with 95% CIs estimated using a bias-corrected and accelerated bootstrap. Net exercise time between sessions 1 and 10 was compared using a Wilcoxon signed-rank test. Statistical significance was defined as 2-sided *P*<.05, including for Bonferroni-adjusted pairwise comparisons. Clinical analyses were exploratory; multiplicity was adjusted within each longitudinal outcome but not across outcomes or for the exploratory correlation analysis. Exercise metrics and safety outcomes were summarized using counts, proportions, and 95% CIs. Wilson score intervals were used for participant-level proportions, participant-cluster bootstrap intervals for session-level and exercise-item–level proportions, and an exact binomial interval for the session-terminating adverse event proportion. For the questionnaire, no inferential tests were performed, and items 1‐11 were summarized as normalized mean ratings (0‐100), calculated as ([mean item rating – 1] / 4)×100.

### Ethical Considerations

The study was approved by the Institutional Review Board of Asan Medical Center (2024‐1159). Written informed consent was obtained from all participants before enrollment after they had received an explanation of the study purpose, procedures, and potential risks. Study data used for analysis were deidentified by removing direct identifiers or replacing them with study-specific codes and were stored in password-protected files on access-restricted institutional systems. Access to the data was limited to the principal investigator and authorized coinvestigators. Each participant received Korean won 100,000 (approximately US $73 based on the exchange rate on August 29, 2026) as compensation for study participation. No study participants are depicted in Figure 1 or Multimedia Appendix 1. The individual shown is a member of the research team from whom separate written consent for publication of the images was obtained.

## Results

### Participants and Their Characteristics

In total, 26 patients were enrolled, completed the baseline evaluation, and initiated the NFA-based in-bed exergaming program (Figure 2). Of these, 2 participants discontinued before the midpoint evaluation because of hospital discharge or pelvic pain following mild trauma. A total of 24 participants completed the midpoint evaluation and 4 subsequently discontinued before the final evaluation owing to hospital discharge or ICU readmission. Ultimately, 20 patients reached session 10 and completed the final evaluation. All discontinuations were judged unrelated to the intervention. Participant-level details of noncompleters, including the clinical context, number of sessions completed, and investigator-assessed intervention relatedness, are provided in Multimedia Appendix 3. All 26 participants contributed baseline data, whereas subsequent analysis sample sizes varied by outcome and assessment time point.

**Figure 2.**
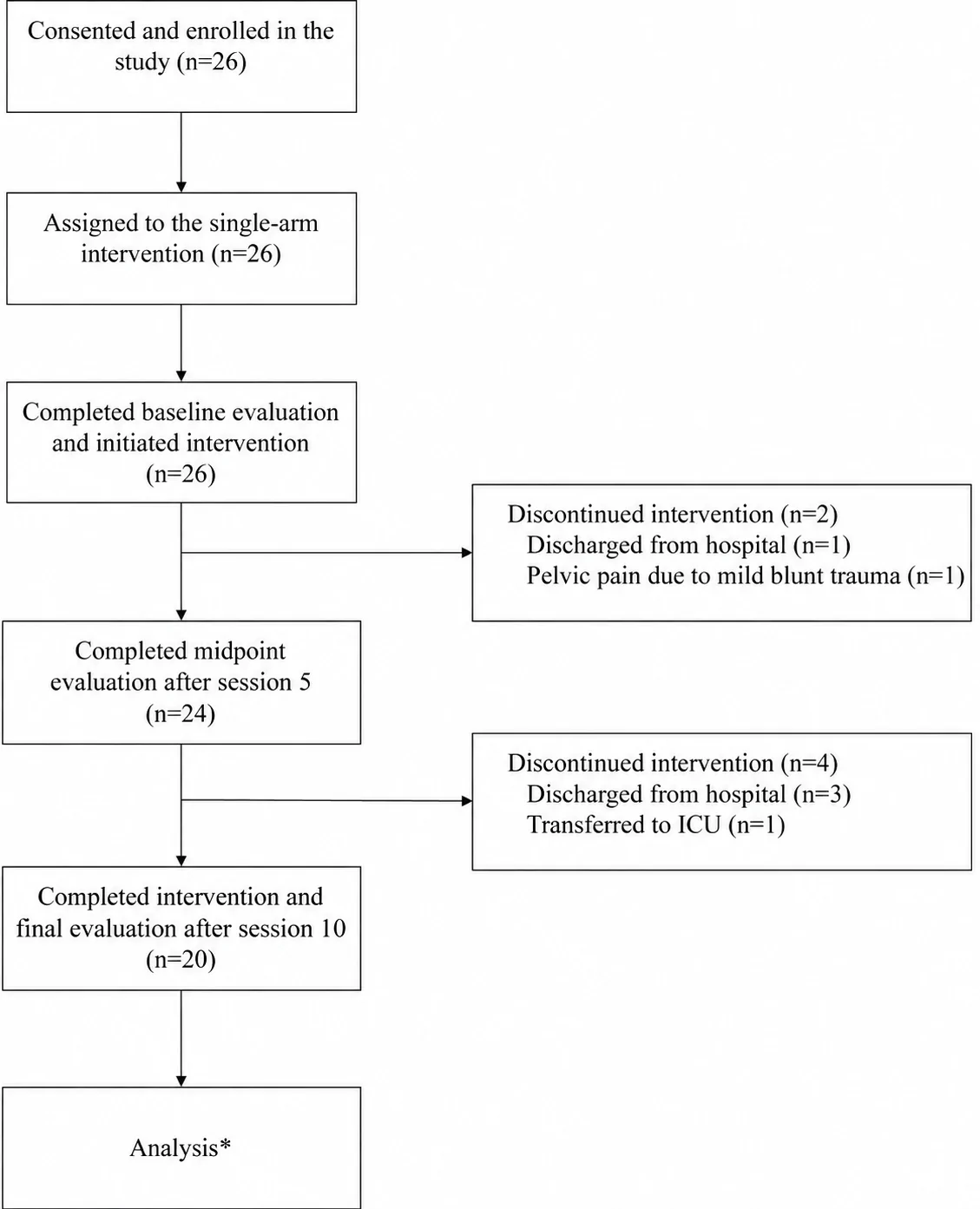
Study flow diagram for a prospective, single-center feasibility study of posttransplant inpatients after intensive care. Flow diagram showing participant enrollment, completion of baseline, midpoint, and final evaluations, and reasons for discontinuation. The study was conducted at Asan Medical Center, Seoul, Republic of Korea, from October 2024 to October 2025. *Sample sizes varied by outcome and type of analysis. Descriptive summaries used available cases, whereas longitudinal tests used outcome-specific complete cases. Exact sample sizes are reported in the corresponding tables and figures. ICU: intensive care unit.

Baseline demographic and clinical characteristics of all enrolled participants, overall and by intervention completion status, are summarized in Table 1. A total of 23 (88.5%) participants underwent liver transplantation, and 3 (11.5%) underwent bilateral lung transplantation. Mean ICU stay and mechanical ventilation (MV) duration were 24.3 (SD 11.0) days and 21.3 (SD 11.6) days, respectively. The first intervention was initiated a mean of 19.6 (SD 13.6) days after ICU discharge. At study entry, participants had marked muscle weakness and mobility limitation, with median MRC-SS, ICUMS, and FAC scores of 38.0 (IQR 30.5-42.0), 6.5 (IQR 3.0-8.0), and 0.5 (IQR 0.0 to 1.0), respectively. Although eligibility permitted FAC scores up to 3, a total of 21 (80.8%) participants were at FAC 0‐1, and none had a score above 2. Most of them required ongoing respiratory support at intervention initiation (tracheostomy cannula: 14/26, 53.8%; bilevel positive airway pressure: 8/26, 30.8%; and/or supplemental oxygen: 14/26, 53.8%). Across all participants, usual PT sessions ranged from 0 to 15, with a mean of 8.3 (SD 4.3); completers received 9.6 (SD 3.7) sessions, compared with 3.8 (SD 3.1) among noncompleters. The corresponding ratios of usual PT sessions to delivered NFA sessions were 0.9 (SD 0.4) overall, 1.0 (SD 0.4) among completers, and 0.7 (SD 0.5) among noncompleters.

**Table 1.** Baseline demographic and clinical characteristics of the participants. Completers were participants who completed all 10 intervention sessions and the final assessment; noncompleters were those who discontinued before completion.

Characteristic	Enrolled (n=26)	Completer (n=20)	Noncompleter (n=6)	
Age (years), mean (SD)	53.0 (12.4)	52.6 (11.7)	54.3 (15.9)	
Sex, n (%)				
Male	14 (53.8)	12 (60)	2 (33.3)	
Female	12 (46.2)	8 (40)	4 (66.7)	
Height (cm), mean (SD)	165.5 (8.7)	166.0 (9.0)	163.6 (8.0)	
Weight (kg), mean (SD)	57.5 (10.4)	57.8 (10.9)	56.7 (9.4)	
BMI (kg/m^2^), mean (SD)	21.1 (4.0)	21.0 (4.0)	21.3 (4.2)	
MMSE-K^a^, mean (SD)	28.2 (1.9)	28.2 (1.9)	28.2 (1.8)	
Baseline blood pressure: systolic/diastolic (mm Hg), mean (SD)	120.2 (17.1)/78.2 (10.2)	121.8 (16.8)/77.3 (9.5)	115.0 (18.8)/81.3 (12.5)	
Pulse rate (beats/min), mean (SD)	86.7 (13.2)	86.4 (14.3)	87.8 (9.6)	
Body temperature (°C), mean (SD)	36.8 (0.4)	36.8 (0.4)	37.2 (0.4)	
Length of hospital stay (days), mean (SD)	84.8 (41.3)	90.9 (42.8)	64.7 (30.4)	
Length of ICU^b^ stay (days), mean (SD)	24.3 (11.0)	24.7 (11.2)	23.2 (10.9)	
Duration of MV^c^ (days), mean (SD)	21.3 (11.6)	22.4 (12.1)	17.8 (9.8)	
APACHE^d^ IV score at ICU admission, mean (SD)	94.1 (18.2)	93.9 (20.5)	94.5 (9.3)	
Time from operation to first intervention (days), mean (SD)	37.0 (16.8)	36.7 (16.9)	38.3 (18.3)	
Time from ICU discharge to first intervention (days), mean (SD)	19.6 (13.6)	18.7 (13.6)	22.7 (14.5)	
MRC-SS^e^, median (IQR)	38.0 (30.5 to 42.0)	40.0 (34.3 to 45.0)	33.0 (30.0 to 36.0)	
ICUMS^f^, median (IQR)	6.5 (3.0 to 8.0)	6.0 (3.0 to 8.0)	7.5 (5.5 to 8.0)	
FAC^g^, median (IQR)	0.5 (0.0 to 1.0)	0.5 (0.0 to 1.0)	0.5 (0.0 to 1.0)	
0, n (%)	13 (50)	10 (50)	3 (50)	
1, n (%)	8 (30.8)	6 (30)	2 (33.3)	
2, n (%)	5 (19.2)	4 (20)	1 (16.7)	
3, n (%)	0 (0)	0 (0)	0 (0)	
Number of usual PT^h^ sessions during intervention, mean (SD)	8.3 (4.3)	9.6 (3.7)	3.8 (3.1)	
Ratio of usual PT sessions to delivered NFA^i^ sessions, mean (SD)	0.9 (0.4)	1.0 (0.4)	0.7 (0.5)	
Respiratory support device at baseline, n (%)				
Tracheostomy cannula	14 (53.8)	11 (55)	3 (50)	
BiPAP^j^ ventilator	8 (30.8)	7 (35)	1 (16.7)	
O_2_ support	14 (53.8)	9 (45)	5 (83.3)	
Reason for ICU admission, n (%)				
Postsurgical	26 (100)	20 (100)	6 (100)	
Bilateral lung transplantation	3 (11.5)	3 (15)	0 (0)	
Liver transplantation	23 (88.5)	17 (85)	6 (100)	
LDLT^k^	16 (61.5)	11 (55)	5 (83.3)	
DDLT^l^	7 (26.9)	6 (30)	1 (16.7)	

a MMSE-K: Korean Version of Mini-Mental State Examination.

b ICU: intensive care unit.

c MV: mechanical ventilation.

d APACHE: Acute Physiology and Chronic Health Evaluation.

e MRC-SS: Medical Research Council Sum Score.

f ICUMS: Intensive Care Unit Mobility Scale.

g FAC: Functional Ambulation Category.

h PT: physical therapy.

i NFA: Nintendo Ring Fit Adventure.

j BiPAP: bilevel positive airway pressure.

k LDLT: living donor liver transplantation.

l DDLT: deceased donor liver transplantation.

In descriptive comparisons, all 6 noncompleters were liver transplant recipients and had a lower median baseline MRC-SS than completers (33.0, IQR 30.0-36.0 vs 40.0, IQR 34.3-45.0), whereas median FAC was identical (0.5, IQR 0.0-1.0 in both groups) and median ICUMS was higher among noncompleters (7.5, IQR 5.5-8.0 vs 6.0, IQR 3.0-8.0). Mean mechanical ventilation duration was shorter among noncompleters (17.8, SD 9.8 vs 22.4, SD 12.1 d). Other baseline differences are shown in Table 1.

### Muscle Strength and Function Measures and HR-QOL

Exploratory clinical outcomes at each time point were summarized using available cases in Table 2, with changes reported as median within-participant differences based on paired observations. Detailed complete-case analyses are provided in Multimedia Appendix 4. The median MRC-SS increased from 38.0 (IQR 30.5-42.0) at baseline to 42.0 (IQR 38.8-48.0) at midpoint and 48.0 (IQR 42.0-48.0) at the final assessment, with a median within-participant change from baseline to final of 8.0 (IQR 2.3-10.3; 95% CI 4.0-10.0). The baseline-to-midpoint and baseline-to-final changes were significant (*P*=.02 and *P*=.004, respectively), whereas the midpoint-to-final change was not significant. The median ICUMS increased from 6.5 (IQR 3.0-8.0) to 8.0 (IQR 6.5-8.0) at midpoint and remained at 8.0 (IQR 8.0-9.0) at final, with a median within-participant change from baseline to final of 2.0 (IQR 1.0-4.3; 95% CI 1.0-3.5). ICUMS showed continued within-participant improvement during both the baseline-to-midpoint and midpoint-to-final periods (*P*=.009 and *P*=.003). FAC and graded SLR also showed significant within-participant changes during the intervention period, whereas bridge-test change was significant only from baseline to final. HGS increased throughout the intervention period, from 10.5 (IQR 6.8-17.1) kg at baseline to 11.8 (IQR 8.6-18.8) kg at midpoint and 15.1 (IQR 10.8-19.3) kg at final, with a median within-participant change from baseline to final of 3.2 (IQR 1.9-4.9; 95% CI 2.1-4.4) kg; however, the increase reached significance only from midpoint to final and from baseline to final. KES also showed an upward trend, although none of the pairwise comparisons reached significance. The number of usual PT sessions was not significantly correlated with baseline-to-final changes in MRC-SS (Spearman ρ=−0.08, 95% CI −0.56 to 0.43; *P*=.75) or ICUMS (Spearman ρ=−0.20, 95% CI −0.61 to 0.29; *P*=.40). Among the 312 scheduled observations included in the missing-data analysis, 35 (11.2%) were missing. The Little test did not reject the hypothesis that the data were missing completely at random (*χ*²_21_=28.45, *P*=.13).

Regarding the EQ-5D-5L measures of HR-QOL (Table 2), the median EQ-5D-5L index increased from 0.429 (IQR 0.193-0.499) at baseline to 0.519 (IQR 0.252-0.659) at midpoint and 0.622 (IQR 0.349-0.731) at final, with a median within-participant change from baseline to final of 0.138 (IQR −0.050 to 0.328; 95% CI −0.008 to 0.309), although the overall change was not statistically significant. The median EQ-VAS increased from 50.0 (IQR 40.0-70.0) at baseline to 60.0 (IQR 50.0-80.0) at midpoint and 65.0 (IQR 50.0-85.0) at final, corresponding to a median within-participant change from baseline to final of 15.0 (IQR 0.0-30.0; 95% CI 5.0-25.0); the baseline-to-final difference was statistically significant. Among the EQ-5D-5L domains, scores in the mobility domain decreased significantly from midpoint to final and from baseline to final.

**Table 2.** Changes in muscle strength, functional measures, and HR-QOL^a^. N denotes all 26 enrolled participants. For time-point summaries, n denotes the number assessed; for change estimates, n denotes the number with paired measurements at the corresponding time points. Changes were calculated as the later value minus the earlier value and are reported as median (IQR), followed by the 95% CI.

Measures category and variable	Baseline		Midpoint		Final		B-M^b^ change		M-F^c^ change		B-F^d^ change	
	n/N (%)	Median (IQR)	n/N (%)	Median (IQR)	n/N (%)	Median (IQR)	n/N (%)	Median change (IQR); 95% CI	n/N (%)	Median change (IQR); 95% CI	n/N (%)	Median change (IQR); 95% CI
Muscle strength and functional measures												
MRC-SS^e^	26/26 (100)	38.0 (30.5 to 42.0)	24/26 (92.3)	42.0 (38.8 to 48.0)	20/26 (76.9)	48.0 (42.0 to 48.0)	24/26 (92.3)	2.5 (0.0 to 7.3); 0.0 to 6.0	20/26 (76.9)	3.5 (0.0 to 6.0); 0.5 to 5.5	20/26 (76.9)	8.0 (2.3 to 10.3); 4.0 to 10.0
ICUMS^f^	26/26 (100)	6.5 (3.0 to 8.0)	24/26 (92.3)	8.0 (6.5 to 8.0)	20/26 (76.9)	8.0 (8.0 to 9.0)	24/26 (92.3)	1.0 (0.0 to 2.0); 0.0 to 1.5	20/26 (76.9)	1.0 (0.0 to 1.3); 0.0 to 1.0	20/26 (76.9)	2.0 (1.0 to 4.3); 1.0 to 3.5
FAC^g^	26/26 (100)	0.5 (0.0 to 1.0)	23/26 (88.5)	1.0 (0.5 to 2.0)	20/26 (76.9)	2.0 (1.0 to 2.0)	23/26 (88.5)	0.0 (0.0 to 1.0); 0.0 to 1.0	20/26 (76.9)	1.0 (0.0 to 1.0); 0.0 to 1.0	20/26 (76.9)	1.0 (0.8 to 1.0); 1.0 to 1.0
Graded SLR^h^	26/26 (100)	2.0 (1.0 to 3.0)	24/26 (92.3)	3.0 (1.0 to 3.0)	20/26 (76.9)	3.0 (2.8 to 3.0)	24/26 (92.3)	0.0 (0.0 to 1.0); 0.0 to 1.0	20/26 (76.9)	0.0 (0.0 to 0.0); 0.0 to 0.0	20/26 (76.9)	0.5 (0.0 to 1.0); 0.0 to 1.0
Bridge test	26/26 (100)	1.0 (1.0 to 2.0)	24/26 (92.3)	2.0 (1.0 to 3.0)	20/26 (76.9)	3.0 (1.0 to 3.0)	24/26 (92.3)	0.0 (0.0 to 1.0); 0.0 to 1.0	20/26 (76.9)	0.0 (0.0 to 0.0); 0.0 to 1.0	20/26 (76.9)	0.5 (0.0 to 1.0); 0.0 to 1.0
HGS^i^ (kg)	26/26 (100)	10.5 (6.8 to 17.1)	24/26 (92.3)	11.8 (8.6 to 18.8)	20/26 (76.9)	15.1 (10.8 to 19.3)	24/26 (92.3)	1.1 (0.0 to 3.0); 0.6 to 2.3	20/26 (76.9)	1.6 (1.1 to 2.5); 1.2 to 2.2	20/26 (76.9)	3.2 (1.9 to 4.9); 2.1 to 4.4
KES^j^ (kg)	15/26 (57.7)	5.4 (4.5 to 7.5)	15/26 (57.7)	8.2 (4.8 to 9.3)	15/26 (57.7)	8.2 (5.7 to 8.9)	14/26 (53.8)	1.4 (0.2 to 2.6); 0.0 to 2.7	13/26 (50)	0.4 (−0.9 to 2.3); −0.9 to 2.3	12/26 (46.2)	2.3 (0.3 to 3.6); 0.0 to 3.9
HR-QOL (EQ-5D-5L)												
EQ-5D index	26/26 (100)	0.429 (0.193 to 0.499)	24/26 (92.3)	0.519 (0.252 to 0.659)	20/26 (76.9)	0.622 (0.349 to 0.731)	24/26 (92.3)	0.033 (−0.021 to 0.181); 0.000 to 0.150	20/26 (76.9)	0.017 (−0.075 to 0.190); −0.046 to 0.130	20/26 (76.9)	0.138 (−0.050 to 0.328); −0.008 to 0.309
Mobility	26/26 (100)	4.0 (3.0 to 5.0)	24/26 (92.3)	3.5 (3.0 to 4.0)	20/26 (76.9)	3.0 (2.0 to 3.3)	24/26 (92.3)	0.0 (−1.0 to 0.0); −1.0 to 0.0	20/26 (76.9)	−1.0 (−1.0 to 0.0); −2.0 to 0.0	20/26 (76.9)	−1.0 (−2.0 to 0.0); −2.0 to 0.0
Self-care	26/26 (100)	5.0 (3.3 to 5.0)	24/26 (92.3)	4.0 (3.0 to 5.0)	20/26 (76.9)	3.0 (2.0 to 4.3)	24/26 (92.3)	0.0 (−1.0 to 0.0); −1.0 to 0.0	20/26 (76.9)	0.0 (−1.0 to 0.0); −1.0 to 0.0	20/26 (76.9)	−1.0 (−2.0 to 0.0); −2.0 to 0.0
Usual activities	26/26 (100)	4.5 (3.0 to 5.0)	24/26 (92.3)	4.0 (3.0 to 5.0)	20/26 (76.9)	3.0 (3.0 to 5.0)	24/26 (92.3)	0.0 (−1.0 to 0.0); 0.0 to 0.0	20/26 (76.9)	0.0 (−0.3 to 0.3); 0.0 to 0.0	20/26 (76.9)	0.0 (−1.3 to 0.3); −1.0 to 0.0
Pain or discomfort	26/26 (100)	2.0 (2.0 to 3.0)	24/26 (92.3)	2.0 (2.0 to 3.0)	20/26 (76.9)	2.0 (2.0 to 3.0)	24/26 (92.3)	0.0 (0.0 to 0.0); 0.0 to 0.0	20/26 (76.9)	0.0 (−1.0 to 1.0); −1.0 to 0.5	20/26 (76.9)	0.0 (−1.0 to 0.3); −1.0 to 0.0
Anxiety or depression	26/26 (100)	2.0 (2.0 to 3.0)	24/26 (92.3)	2.0 (1.0 to 3.0)	20/26 (76.9)	2.0 (1.0 to 2.3)	24/26 (92.3)	0.0 (−0.3 to 0.0); 0.0 to 0.0	20/26 (76.9)	0.0 (−1.0 to 0.0); −1.0 to 0.0	20/26 (76.9)	−1.0 (−1.0 to 0.0); −1.0 to 0.0
EQ-VAS	26/26 (100)	50.0 (40.0 to 70.0)	24/26 (92.3)	60.0 (50.0 to 80.0)	20/26 (76.9)	65.0 (50.0 to 85.0)	24/26 (92.3)	10.0 (0.0 to 20.0); 5.0 to 15.0	20/26 (76.9)	10.0 (−6.3 to 20.0); −2.5 to 20.0	20/26 (76.9)	15.0 (0.0 to 30.0); 5.0 to 25.0

a HR-QOL: health-related quality of life.

b B-M: baseline to midpoint.

c M-F: midpoint to final.

d B-F: baseline to final.

e MRC-SS: Medical Research Council Sum Score.

f ICUMS: Intensive Care Unit Mobility Scale.

g FAC: Functional Ambulation Category.

h SLR: straight leg raise.

i HGS: handgrip strength.

j KES: knee extensor strength.

### Exercise Metrics and Adverse Events

Table 3 summarizes participant retention and session- and exercise-item–level metrics. Of the 26 enrolled participants, 20 completed the final assessment, corresponding to a retention rate of 76.9% (95% CI 57.9%-89%). Across all enrolled participants, 227 of the 260 (87.3%; 95% CI 76.9%-95.8%) protocol-planned sessions were delivered, and 223 were completed, corresponding to 85.8% of all protocol-planned sessions (95% CI 75%-95%). Of these delivered sessions, 223 (98.2%; 95% CI 95.8%-100%) were completed, whereas 4 were interrupted because of fatigue. No session-terminating adverse events (0/227, 0%; 95% CI 0%-1.6%) were observed during 227 delivered sessions. Of the prescribed items, 2103 (97.3%; 95% CI 95.2%-98.9%) were attempted. Among the attempted items, 31 (1.5%; 95% CI 0.7%-2.5%) were interrupted after initiation, primarily because of transient fatigue (n=23), followed by upper-arm pain (n=8). The completion rate was highest for upper-extremity exercises (97.2%), followed by lower-extremity exercises (96.8%), and was lowest for trunk exercises (94.4%). Multimedia Appendix 5 provides detailed distributions of prescribed and completed items and completion rates by target body region and individual exercise type.

**Table 3.** Participant retention, intervention delivery, and prescribed exercise-item completion during the 10-session in-bed NFA^a^ program among the 26 enrolled participants. Outcomes are presented at the participant, session, and exercise-item levels to characterize retention, intervention delivery, and prescribed exercise-item completion, respectively, together with recorded reasons for interruption and noncompletion. Values at the session and exercise-item levels are aggregated across the participants over the intervention period. Total planned sessions were calculated as 10 sessions for each of the 26 enrolled participants. Body part-specific interruption percentages used the number of attempted items within the corresponding targeted body part.

Analysis level and variable	Values	
	n/N (%)	95% CI
Participant level		
Program retention	20/26 (76.9)	57.9 to 89
Session level		
Total planned sessions	260^b^	N/A^c^
Delivered sessions	227/260 (87.3)	76.9 to 95.8
Overall protocol-planned session completion	223/260 (85.8)	75 to 95
Delivered-session completion	223/227 (98.2)	95.8 to 100
Interrupted sessions	4/227 (1.8)	0 to 4.2
Reasons for interruption		
Session-terminating adverse event	0/227 (0)	0 to 1.6
Fatigue	4/227 (1.8)	0 to 4.2
Exercise item level		
Total prescribed exercise items	2161^b^	N/A
Attempted exercise items	2103/2161 (97.3)	95.2 to 98.9
Completed exercise items	2072/2161 (95.9)	93.4 to 97.9
Interrupted attempted items	31/2103 (1.5)	0.7 to 2.5
Upper extremity	7/588 (1.2)	0.3 to 2.5
Reasons for interruption		
Due to upper arm pain	4/588 (0.7)	0 to 1.9
Due to fatigue	3/588 (0.5)	0 to 1.2
Lower extremity	14/642 (2.2)	0.8 to 3.8
Reasons for interruption		
Due to fatigue	14/642 (2.2)	0.8 to 3.8
Trunk	10/873 (1.1)	0.2 to 2.3
Reasons for interruption		
Due to fatigue	6/873 (0.7)	0.1 to 1.5
Due to upper arm pain	4/873 (0.5)	0 to 1.5

a NFA: Nintendo Ring Fit Adventure.

b n value.

c N/A: not applicable.

Figure 3 presents complete-case data from the 20 participants who completed the final assessment. The aggregate numbers of prescribed and completed exercise items increased during the early sessions and subsequently stabilized, while the failure rate generally decreased over time (Figure 3A). Consistent with Figure 3A, participant-level distributions showed that prescribed exercise-item counts stabilized earlier, whereas completed counts progressively approached them, narrowing the gap across sessions (Multimedia Appendix 6). Median net exercise time increased from 283.0 (IQR 246.5-380.0) seconds in session 1 to 463.0 (IQR 326.0-622.8) seconds in session 10, with a median within-participant increase of 159.0 (IQR 35.5-296.3; 95% CI 81.0-284.0 seconds; *P*<.001; Figure 3B).

**Figure 3.**
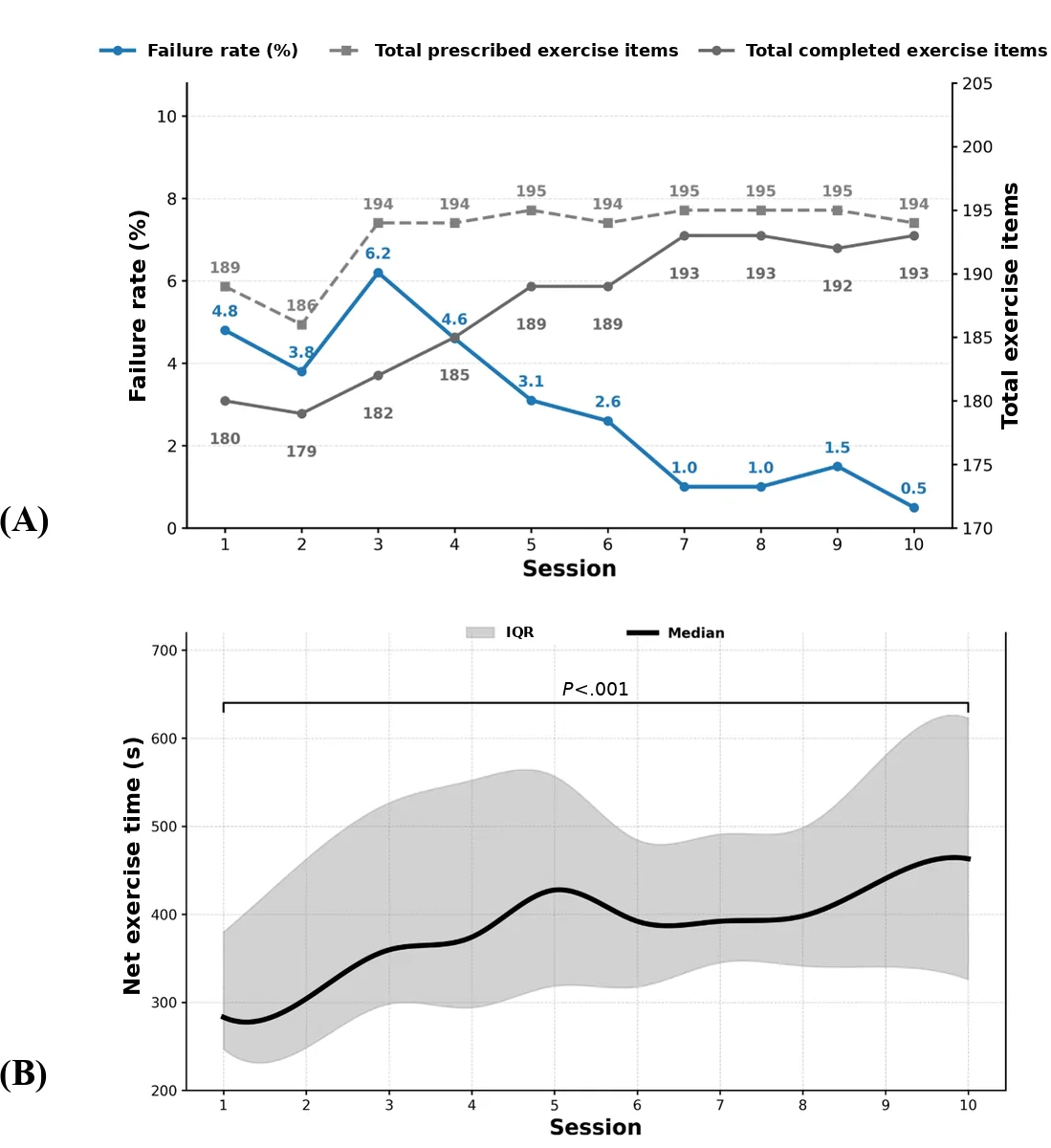
Trends in prescribed and completed exercise items, failure rate, and net exercise time across 10 intervention sessions. Data were obtained from 20 participants who completed all 10 intervention sessions (n=20 per session). (A) Changes in the aggregate number of prescribed and completed exercise items pooled across all participants. The blue line indicates the failure rate (%), defined as the proportion of prescribed exercises that were not completed. Prescribed exercises were classified as not completed if they were not attempted or were interrupted. The gray dashed line indicates the total number of prescribed exercise items, whereas the gray solid line indicates the total number of completed exercise items for each session. (B) Changes in net exercise time (s), defined as the actual duration of exercise performance excluding rest periods. Data are presented as the median (solid line) with IQR (shaded area). Sessions 1 and 10 were compared using a 2-sided Wilcoxon signed-rank test. The net exercise time was significantly higher in session 10 than in session 1 (463.0, IQR 326.0-622.8 vs 283.0, IQR 246.5-380.0 seconds) with a median within-participant increase of 159.0 (IQR 35.5-296.3; 95% CI 81.0-284.0; *P*<.001) seconds.

Among the 19 participants with complete HR data at all 3 assessments, neither resting nor peak HR differed significantly across assessments (*P*=.29 and *P*=.10, respectively; Figure 4A), and the highest peak HR recorded across the 3 assessments was 132 beats/min. The median absolute increase from resting to peak HR was 22 (IQR 16-36), 21 (IQR 17.5-30.5), and 24 (IQR 15.5-35.5) beats/min, respectively, with no significant difference across assessments (*P*=.50; Figure 4B).

**Figure 4.**
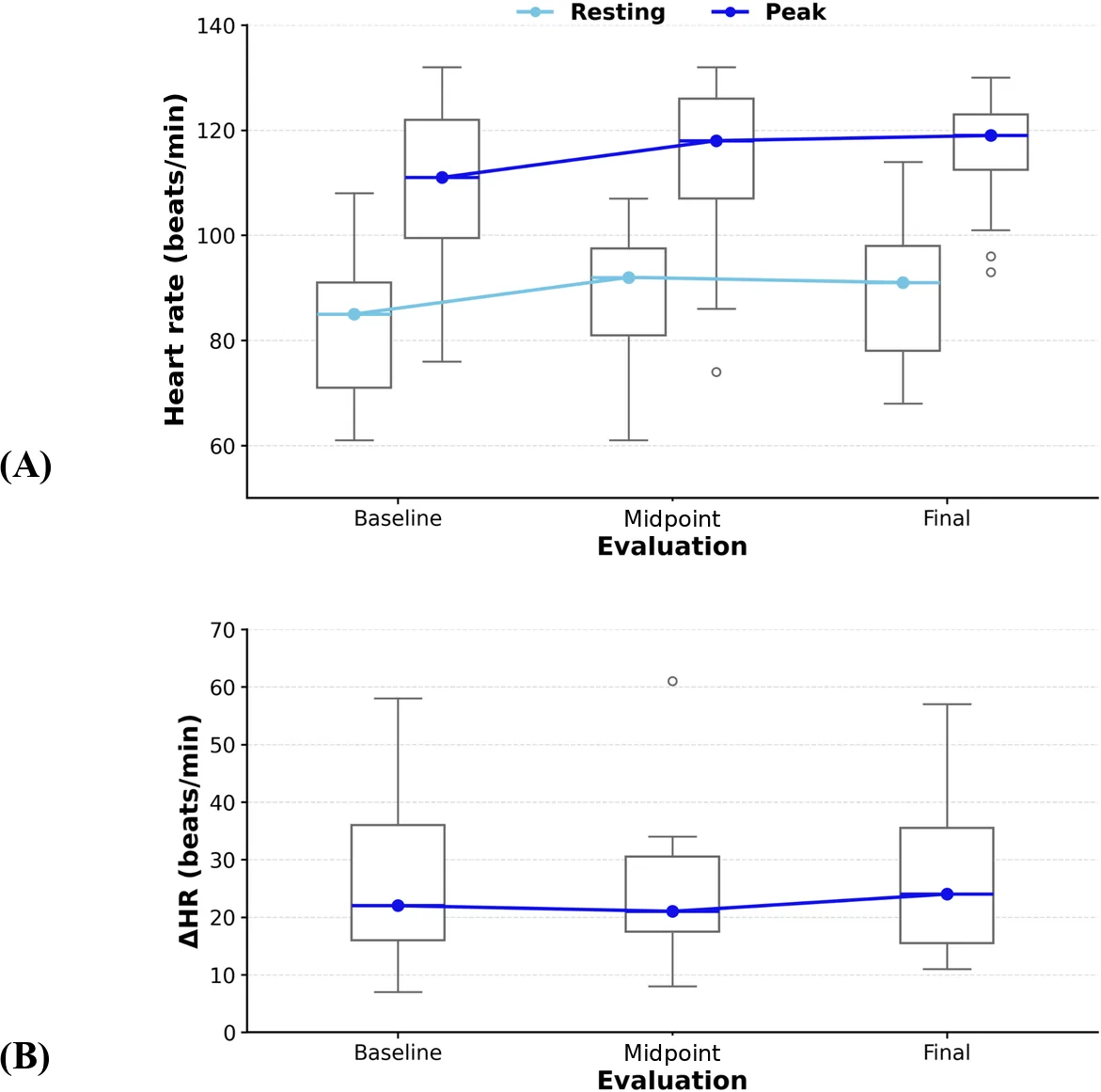
Heart rate responses across assessments. Box-and-whisker plots show complete-case data from the same 19 participants at baseline, midpoint, and final assessments (n=19 at each assessment). One intervention completer with no peak HR data at any assessment was excluded. Center lines indicate medians, and overlaid lines connect median values to illustrate temporal trends. (A) Resting and peak HR. (B) Peak-resting HR difference (ΔHR), calculated as peak HR minus resting HR. Median (IQR) ΔHR values were 22 (16-36), 21 (17.5‐30.5), and 24 (15.5‐35.5) beats/min at baseline, midpoint, and final assessments, respectively (Friedman test, *P*=.50). HR: heart rate.

### User Experience and Satisfaction

Questionnaire responses were available from 19 of the 24 (79.2%) participants who completed the midpoint assessment and 18 of the 20 (90%) who completed the final assessment. Responses to items 1‐11 were concentrated in the “agree” and “strongly agree” categories at both assessments, and all normalized mean item ratings were within the upper quarter of the 0‐100 scale (Figure 5A). The “willingness to continue” item had the lowest normalized mean rating of 78.9 (SD 28.0) at midpoint, but the rating was higher at 86.1 (SD 21.4) at final. Most respondents rated exercise intensity as “appropriate” at both midpoint (14/19, 73.7%) and final (14/18, 77.8%; Figure 5B). Four (21.1%) respondents rated the intensity as “difficult” at midpoint, whereas only 2 (11.1%) respondents gave ratings above “appropriate” at final.

**Figure 5.**
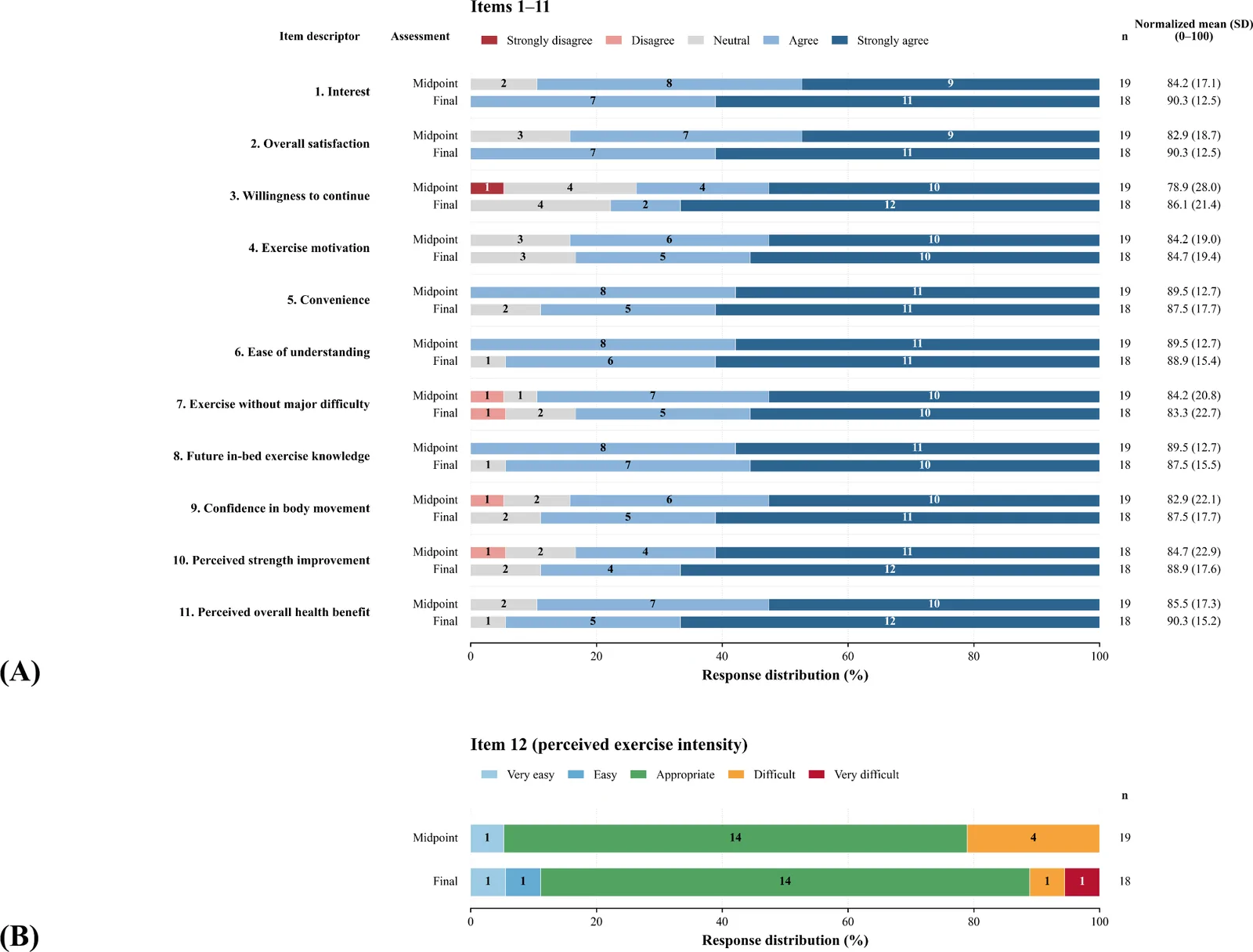
Item-level response distributions for the study-specific satisfaction questionnaire. Distributions of responses to items 1‐12 at the midpoint and final assessments. Each 100% stacked horizontal bar shows the distribution of response categories among participants with a nonmissing response to the corresponding item at each assessment. Numbers within bar segments indicate response counts, and n indicates the number of participants with a nonmissing response to the corresponding item at that assessment. (A) Distributions of responses to items 1‐11. Normalized mean (SD) item rating on a 0‐100 scale, calculated as ([mean item rating – 1] / 4) ×100. Higher values indicate stronger agreement with the corresponding statement. (B) Distribution of responses to the perceived exercise intensity item.

## Discussion

### Principal Findings

To the best of our knowledge, this is the first study to evaluate a commercially available exergame on a general ward as an in-bed rehabilitation intervention for posttransplant patients with marked functional impairment after ICU care. Participant retention and delivery of protocol-planned sessions were generally favorable, with high completion among delivered sessions. Most prescribed exercise items were completed, supporting item-level implementation fidelity. No session-terminating adverse events were observed. Questionnaire responses were concentrated in the “agree” and “strongly agree” categories, and all normalized item ratings were in the upper quarter of the scale. Overall, the program showed favorable operational feasibility, with supportive findings for observed short-term safety, tolerability, and acceptability under therapist supervision.

Importantly, the study population comprised high-acuity posttransplant recipients with profound post-ICU functional impairment. Most participants were either nonfunctional ambulators or required continuous physical assistance for ambulation at baseline. Prolonged MV courses and ICU stays [43] were characteristic of the study population, both of which are associated with worse physical function [44,45]. Muscle weakness was similarly pervasive, with most participants meeting the criteria for ICU-AW [46], while Acute Physiology and Chronic Health Evaluation IV scores reflected substantial acute illness severity at ICU admission. This profile differs from those of populations in other exergaming studies, which have largely focused on community-dwelling older adults [33,35], neurologic rehabilitation cohorts [27], or hospitalized older adult patients [34] with relatively preserved function. Even in critical-care–related exergaming studies, functional reserve was generally greater. For example, in a study by Kho et al [32], walking was the highest activity level in half of the participants, and the length of ICU stay was shorter (median 8.5, IQR 2.8-28.8 vs mean 24.3, SD 11.0 d). In Wang et al [25], median baseline MRC-SS scores were 46‐48; approximately one-third of participants met the criteria for ICU-AW, compared with an MRC-SS of 38.0 (IQR 30.5-42.0) and a higher ICU-AW prevalence in this study. For critical-care and posttransplant inpatients with ICU-AW and severe deconditioning, maintaining adequate physical activity during hospitalization is essential to support functional recovery and limit the harms of prolonged bed rest [44]. However, ongoing respiratory care needs and other medical complexities may preclude gym-based rehabilitation and shift care toward bedside-only delivery [16,17,47]. Within this setting, however, profound weakness limits patients to a narrow and repetitive range, while fatigue and psychological barriers [10,11,13-15,48] further hinder sustained participation. Introducing exergaming into bedside rehabilitation may help sustain engagement through goal-directed tasks and immediate feedback [19,23,49], particularly when participation is constrained by profound functional impairment or low motivation [11,13,14]. Consistent with this potential mechanism, participants generally gave favorable self-reported ratings on the study-specific questionnaire items assessing interest in exercising with the device and motivation to exercise. Accordingly, this study population represented a particularly challenging clinical context for implementing in-bed exergaming.

As noted above, no session-terminating adverse events were observed despite the substantial deconditioning of the study participants and ongoing respiratory support required by more than half at intervention initiation. Tolerability was also favorable, with infrequent and manageable exercise-related interruptions. Fatigue was the predominant limiting symptom among interrupted exercise items, consistent with another study on exergaming feasibility for early ICU mobilization [50] and with a report that highlighted fatigue as a common issue following solid-organ transplantation [15]. Upper-limb pain was another limiting factor and occurred during both upper-extremity and trunk exercises, which required sustained Ring-Con manipulation and coordinated upper-body movements. These symptoms were predominantly localized to the shoulder or proximal arm, aligning with frequent reports of post-ICU shoulder complications, such as adhesive capsulitis and restricted range of motion [51,52]. However, these transient symptoms were successfully managed with brief rest or task modification without clinical deterioration. Regarding observed short-term safety, although not a definitive safety threshold, the upper HR boundary of 130 beats/min in ICU mobilization criteria provides a conservative reference for the highest peak HR of 132 beats/min observed in this study [53]. This criterion was developed for patients still in the ICU and therefore serves as a conservative benchmark for participants already on general wards. Together with the absence of session-terminating adverse events, these findings indicate that no serious safety concerns were observed during short-term delivery under therapist supervision.

In addition to the overall feasibility findings summarized above, among participants who completed all sessions, median net exercise time increased from session 1 to session 10, an approximately 64% increase in the group median. This pattern suggests that the program could be adjusted as participants tolerated more exercise over time, providing additional evidence of operational feasibility. Regarding participant acceptance, no study discontinuation, session interruption, or exercise-item interruption was attributed to participant refusal. The generally higher item ratings at the final assessment may reflect greater familiarity and acceptance following an initial period of adaptation to the exergame, consistent with the exercise-performance trends. Although good acceptability was also reported in another NFA-based study without individualized prescription [33], the satisfaction and engagement metrics were higher in our study. This difference may relate to the structured nature and personalization of the program, which allowed exercise selection and dosing to be adjusted according to the daily clinical status of each patient, thereby maintaining an appropriate level of challenge. This interpretation is supported by the high exercise-item completion rate and perceived intensity ratings centered on the “appropriate” level. Given the substantial functional and psychological barriers to sustained exercise participation in posttransplant patients with ICU-AW, including helplessness, fear of movement, and low self-efficacy [10,11,14,15], the observed session-level adherence and acceptability results are encouraging.

Among participants who completed the final evaluation, longitudinal trends in completed exercise-item counts and net exercise time paralleled the observed functional changes during the early-to-mid intervention period, particularly through session 6, with initial fluctuations during sessions 1‐3 possibly reflecting an early familiarization period. This early-to-mid increase coincided with clear within-participant increases at the midpoint, including statistically significant gains in MRC-SS, ICUMS, and graded SLR. The individualized prescription may have supported both early adaptation and subsequent progression by allowing exercise selection and dosing to be adjusted in line with observed improvements in strength and mobility, thereby aligning completed exercise volume with evolving functional capacity. In later sessions, completed exercise-item counts plateaued, whereas the net exercise time increased toward the end of the program, which may have resulted from additional repetitions within selected items, although repetition counts were not recorded. This descriptive temporal alignment provides an exploratory account of how exercise exposure evolved alongside the observed functional changes during the intervention period.

Modern rehabilitation technologies, particularly purpose-built exergaming systems, can provide task-specific practice, multimodal feedback, quantifiable performance targets, and adjustable difficulty, allowing exercise demands to be matched more closely to patient ability [20,21,30]. However, their development and routine implementation can require substantial resources, including acquisition, maintenance, and technical support [54]. Commercially available off-the-shelf exergames are more accessible, but their entertainment-oriented, predetermined movements and fixed progression structures may not adequately accommodate patients’ clinical goals or functional capacity [20,55], a limitation that may be particularly relevant for severely deconditioned, medically complex inpatients. In this study, the NFA custom mode provided a pragmatic compromise, retaining the accessibility of a commercial platform while enabling exercises to be selected, sequenced, and dosed according to each patient’s daily clinical status and functional capacity. Another distinguishing feature of our study was the incorporation of trunk and lower extremity exercises as a substantial component of an in-bed program. Previous in-bed exergaming studies predominantly emphasized upper extremity training, whereas meaningful lower-limb engagement in other protocols often required standing [30-32,34,50]. Given that proximal stability and lower limb strength are foundational for mobilizing patients with ICU-AW [56,57], extending the exercise repertoire to these regions in a bed-based setting is clinically relevant, particularly for this population with marked functional impairment.

Although the NFA provided a practical compromise between bespoke rehabilitation software and a conventional off-the-shelf exergame, several pragmatic observations and limitations may inform the future development and clinical use of exergames. First, prescribed and completed exercise-item counts plateaued during later sessions despite a modest late increase in net exercise time. The custom mode’s 10-item limit may have contributed to this plateau; together with the absence of exercise-specific difficulty adjustment, it may also have limited the systematic application of progressive loading. Exergame platforms incorporating a broader exercise range and adjustable task difficulty may better accommodate progression across changing functional levels. Second, trunk exercises comprised more than half of the study library but had the lowest completion rate. The Back Press and Overhead Side Bend were among the least frequently prescribed. In contrast, the upper- and lower-extremity exercises were prescribed more consistently, and each achieved a completion rate above 95%. Future libraries should include trunk exercises better suited to bed-based delivery while expanding the variety and gradability of limb-focused options. Third, although sessions were planned within a typical 30-minute ward-based therapy window, delivery was occasionally prolonged, mainly by intercurrent bedside care such as suctioning, suggesting that this time window may leave limited flexibility for concurrent clinical care in medically complex inpatients. Finally, resource and workflow implications warrant particular attention. Separate from delays related to intercurrent clinical care, device setup and calibration were required for every session and occasionally contributed to prolonged delivery. Safety monitoring, exercise instruction, and manual assistance remained necessary. In many inpatient settings, staffing constraints and restrictive reimbursement can limit the availability of bedside therapy, leaving severely impaired patients with few opportunities for active movement outside scheduled sessions [12,16-18]. However, given the support required in this study and the absence of formal resource-use or cost evaluation, this approach cannot be assumed to reduce resource use relative to usual bedside PT or to address these constraints; these potential benefits require objective evaluation. Nevertheless, the potential for familiarization with in-bed exergaming to increase activity beyond scheduled therapy [24], together with the preliminary evidence of deliverability and the observed short-term tolerability under monitoring in this study, supports further evaluation of both changes in activity behavior and less resource-intensive delivery models. Future studies should examine whether, after initial familiarization, one-to-one supervision can be reduced or shifted toward one-to-many delivery, and whether this approach can increase patients’ activity beyond scheduled therapy and thereby expand exercise opportunities for this population. Addressing these questions will require wearable sensor–based measurement of activity and sedentary time, together with focused implementation outcomes covering device setup and technical failures, staff time and workload, and direct costs, preferably in comparison with conventional bedside exercise.

### Limitations

This study had several methodological limitations. First, the single-center, single-arm design without a concurrent comparator and the short intervention period with no postintervention follow-up limited causal inference and assessment of sustained changes. The observed functional changes may reflect natural recovery and concurrent usual PT rather than an independent effect of the NFA-based intervention. Second, attrition and missing data may have affected the longitudinal findings. Although the Little test did not reject the missing completely at random hypothesis, its limited power in this small sample left the missing-data mechanism uncertain. In particular, KES was available for only a subset of participants because reliable measurements were often difficult to obtain in participants with marked weakness, raising the possibility of nonrandom missingness and bias toward stronger participants. Complete-case and completer-only analyses may be affected by selection or attrition bias, while across-session patterns may also reflect therapist learning. Third, physiological responses were characterized using HR measurements obtained only at baseline, midpoint, and final assessments. HR responses may also have been influenced by medication use and altered autonomic or chronotropic function after critical illness and transplantation [58]. Other relevant measures, including oxygen saturation and dyspnea, were not systematically recorded across sessions. Safety outcomes also focused on events meeting the criteria for session termination and may not have captured less severe or transient symptoms. Fourth, this study evaluated selected aspects of operational feasibility rather than the full range of implementation domains. The absence of a screening denominator prevented evaluation of recruitment efficiency, and prespecified feasibility progression thresholds were not established. Technical reliability was not formally evaluated, while item-level completion did not capture repetition counts or the extent of manual assistance. Nonetheless, operational feasibility was quantified at the participant, session, and exercise-item levels. Findings may also not generalize to patients with greater clinical instability or cognitive or sensory limitations that precluded interaction with the exergame, or to other post-ICU populations. The inclusion of both liver and bilateral lung transplant recipients also introduced clinical heterogeneity. The exploratory clinical outcomes may have been affected by unblinded assessment and variation in testing procedures, including manual rather than external stabilization during KES testing. The study-specific SLR and bridge grading systems had not been formally validated, and multiplicity was not adjusted across outcomes. In addition, the satisfaction questionnaire was nonvalidated and nonanonymous, and its wording and administration by treating therapists may have introduced social desirability and response bias. Finally, the small sample limited the precision of feasibility and safety estimates, as reflected by the wide 95% CI for final-assessment retention, and precluded meaningful subgroup analyses.

### Conclusions

In conclusion, when delivered under therapist supervision, the individually prescribed in-bed NFA program for posttransplant inpatients after ICU care showed favorable indicators of operational feasibility, including high delivered-session adherence and item-level implementation fidelity. Within the same supervised setting, exercise-item interruptions were infrequent and generally managed through simple modification or substitution of exercise items, and no session-terminating adverse events were observed, suggesting a low apparent short-term risk profile. Participant satisfaction was also generally favorable. By combining the accessibility of a commercial off-the-shelf exergame with individualized exercise prescription, the program extended the application of bedside exergaming to posttransplant inpatients with substantial functional impairment after ICU care who had not achieved independent ambulation. The resulting implementation data and adapted exercise framework may inform the practical application and further evaluation of similar bedside programs. Future controlled studies should determine clinical benefit and broader safety, including less severe or transient exercise-related symptoms, and evaluate how platform and protocol refinements affect the resource requirements and scalability.

## Supplementary material

10.2196/95037 Multimedia Appendix 1 In-bed exergaming exercise library and clinical implementation. Detailed overview of the 13 exercise types prescribed in the in-bed exergaming program, categorized by target body region and primary therapeutic focus. For each exercise, an illustration based on a Nintendo Ring Fit Adventure reference image depicting the standard exercise posture is shown alongside a demonstration of the bedside-adapted version. The adaptation retained the primary target body region but was not considered biomechanically equivalent to the standard exercise.

10.2196/95037 Multimedia Appendix 2 Satisfaction questionnaire. Items 1-11 use a 5-point Likert scale (1=strongly disagree to 5=strongly agree). Item 12 assesses perceived intensity (1=very easy to 5=very difficult). The final item is an open-ended question.

10.2196/95037 Multimedia Appendix 3 Detailed description of noncompleters.

10.2196/95037 Multimedia Appendix 4 Statistical analysis of change in muscle strength, functional measures, and health-related quality of life. Unless otherwise specified, statistical analyses were restricted to participants with complete data at all assessment time points (n=20). Statistical significance for overall change was tested using the Friedman test. Post hoc pairwise comparisons were performed only when the overall Friedman test was statistically significant, using 2-sided Wilcoxon signed-rank tests. Pairwise *P* values were Bonferroni-adjusted for the 3 comparisons within each outcome; adjusted *P* values <.05 were considered statistically significant. *P<.05, **P<.01, ***P<.001. KES was analyzed for participants with complete data (n=12).

10.2196/95037 Multimedia Appendix 5 Detailed frequency distribution of prescribed and completed exercise items. Exercise item-level outcomes describe the distribution and completion of individualized exercise item prescriptions across target body regions and exercise types. Values are presented as n (%) unless otherwise indicated. Percentages in the prescribed and completed columns were calculated using all prescribed items (N=2161) and all completed items (N=2072), respectively. Exercise-specific completion rates were calculated using the number of prescribed items for the corresponding exercise type as the denominator.

10.2196/95037 Multimedia Appendix 6 Participant-level frequency distributions of prescribed and completed exercise items across 10 intervention sessions. Data were obtained from 20 participants who completed all 10 intervention sessions (n=20 per session). Each cell indicates the number of participants with the corresponding exercise item count; darker colors indicate a greater number of participants. Blank cells indicate that no participant had the corresponding count. (A) Number of prescribed exercise items per participant at each session. (B) Number of completed exercise items per participant at each session.

10.2196/95037 Checklist 1 TIDieR Checklist.

## Abbreviations

FAC: Functional Ambulation Category
HGS: handgrip strength
HR: heart rate
HR-QOL: health-related quality of life
ICU: intensive care unit
ICU-AW: intensive care unit-acquired weakness
ICUMS: Intensive Care Unit Mobility Scale
KES: knee extensor strength
MRC-SS: Medical Research Council Sum Score
NFA: Nintendo Ring Fit Adventure
PT: physical therapy
SLR: straight leg raise
TIDieR: Template for Intervention Description and Replication
