# Supplementary material for: Individually Prescribed In-Bed Exergaming Using Nintendo Ring Fit Adventure in Posttransplant Inpatients With Marked Functional Impairment After Intensive Care: Prospective Single-Arm Feasibility Study
Source: JMIR Serious Games. 2026 Oct 20;14:e95037. doi: 10.2196/95037 (PMC13684350; doi:10.2196/95037)
Supplement: Supplementary file 1 [file games-v14-e95037-s001.pdf]

Targeted Body Region	Exercise	NFA Reference Image	In-Bed Modification	Main Therapeutic Focus
Upper extremity	Bow pull	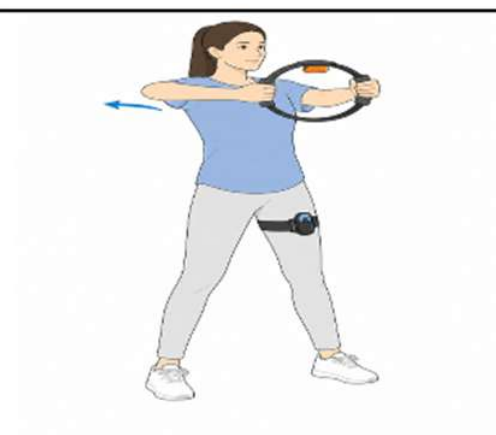	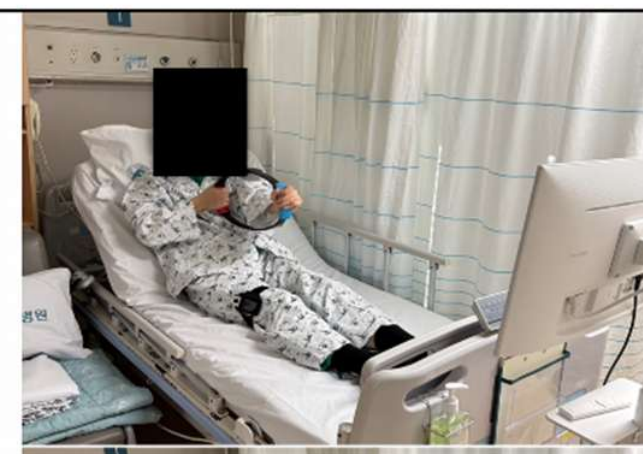	Bilateral upper-extremity strengthening
	Overhead arm spin	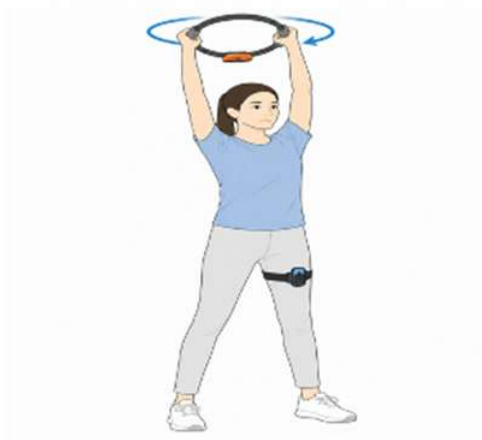	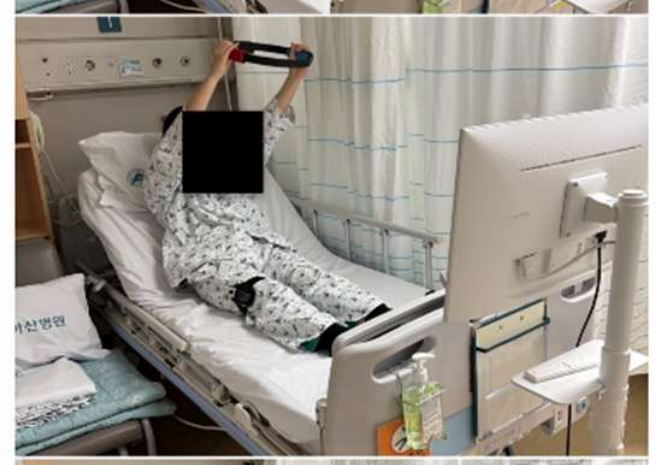	Bilateral upper-extremity flexibility and overhead arm coordination
	Overhead press	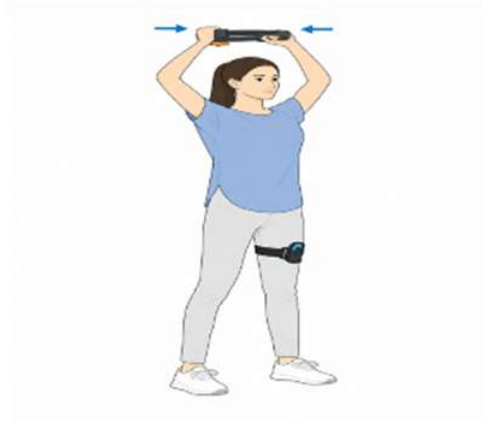	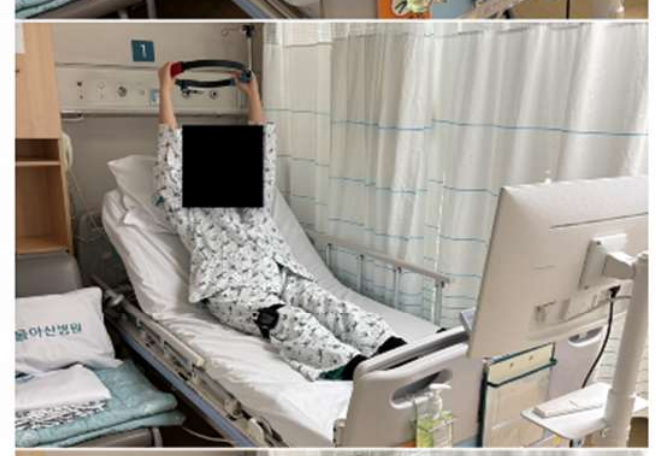	Bilateral upper-extremity strengthening
Lower extremity	Thigh press	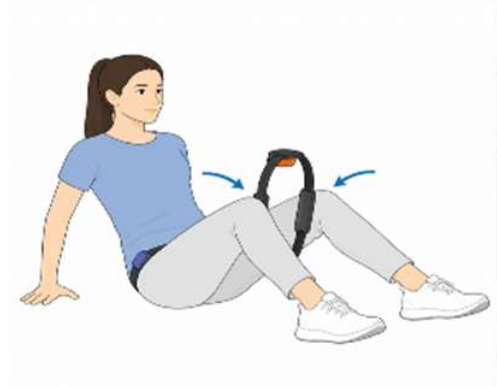	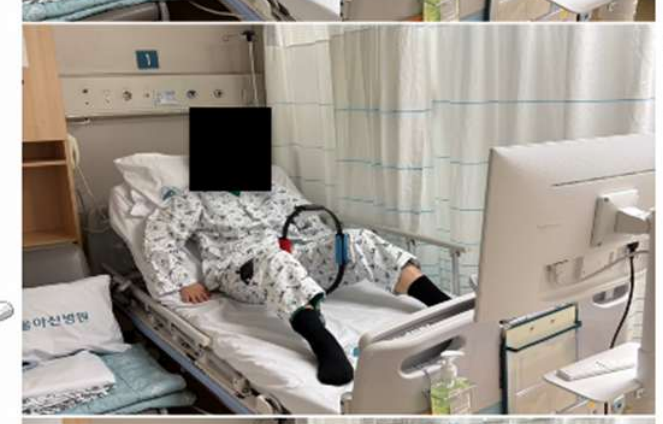	Bilateral lower-extremity strengthening (hip adductors)
	Knee to chest	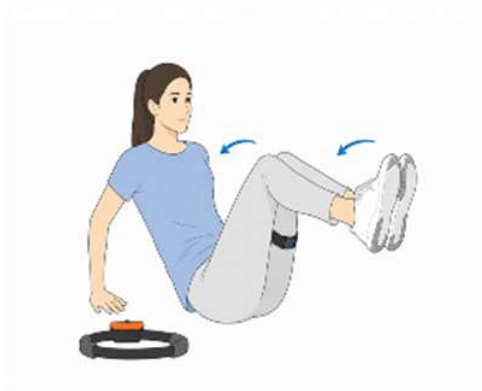	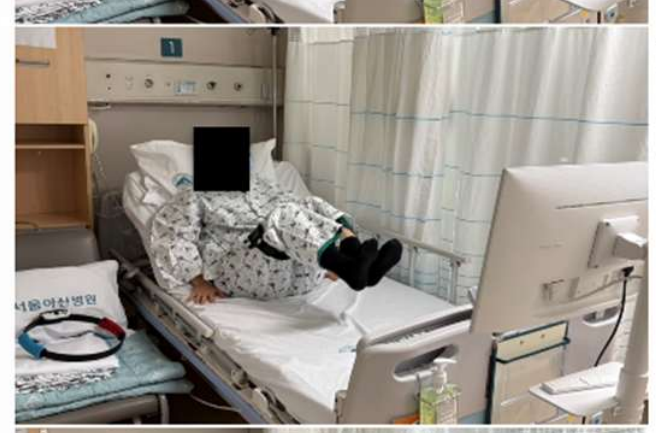	Bilateral lower-extremity strengthening, and coordination with core control
	Hip lift	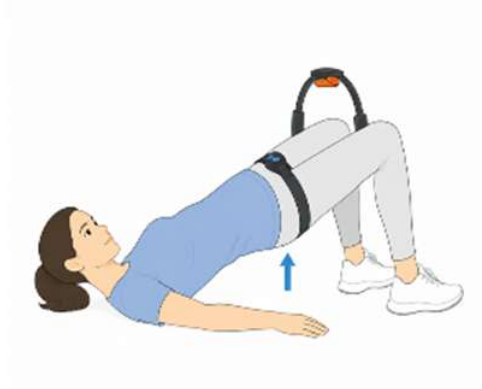	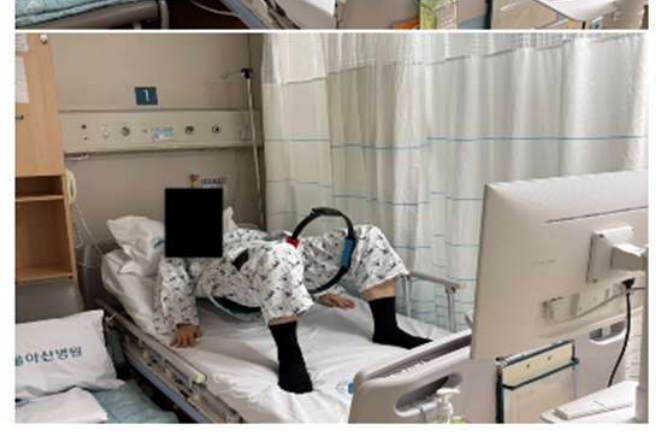	Bilateral lower-extremity strengthening and coordination with core control

Targeted Body Region	Exercise	NFA Reference Image	In-Bed Modification	Main Therapeutic Focus
Trunk	Front press	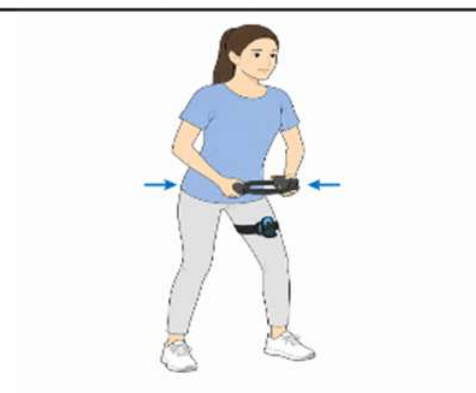	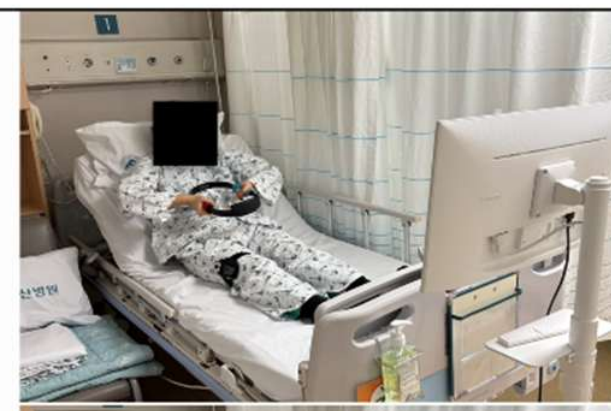	Trunk strengthening (anterior) with bilateral upper-extremity coordination
	Russian twist	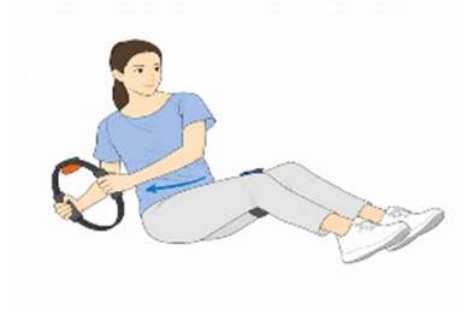	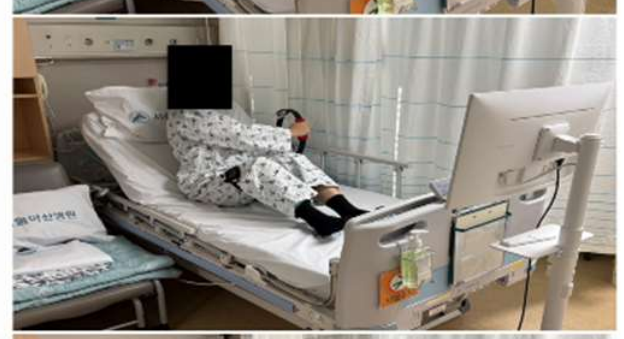	Trunk control and flexibility
	Seated forward press	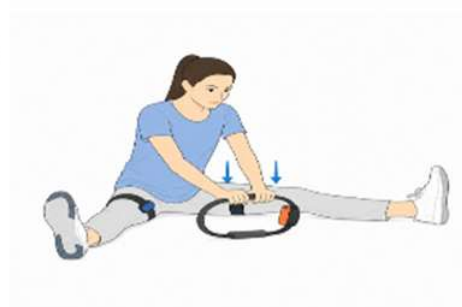	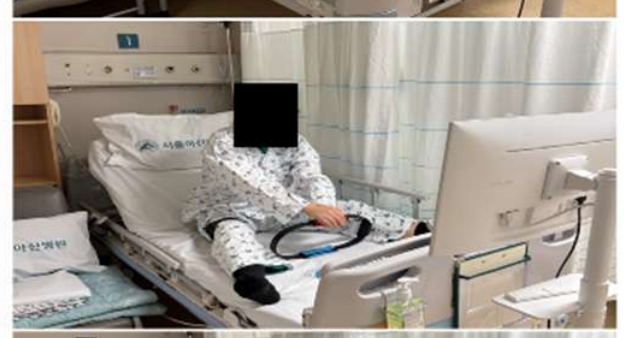	Trunk strengthening (anterior) with bilateral upper-extremity coordination
	Seated ring raise	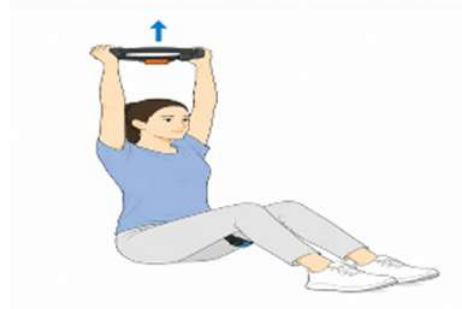	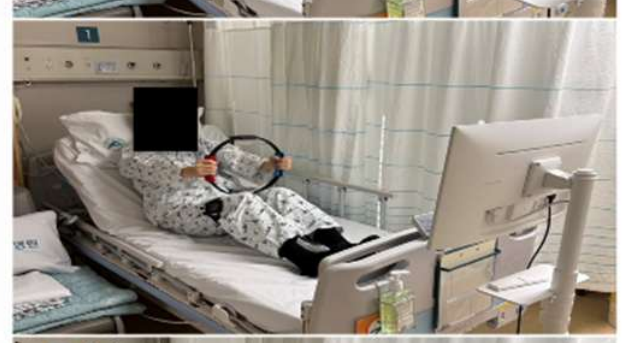	Trunk control with bilateral upper-extremity coordination
	Overhead bend	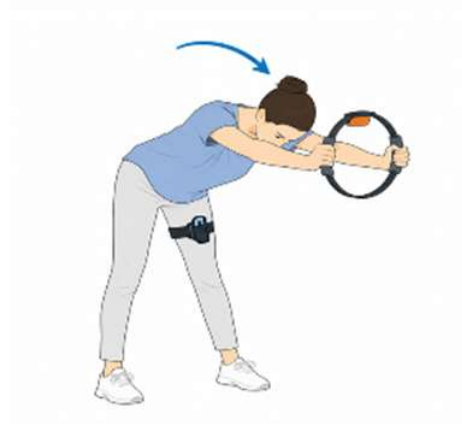	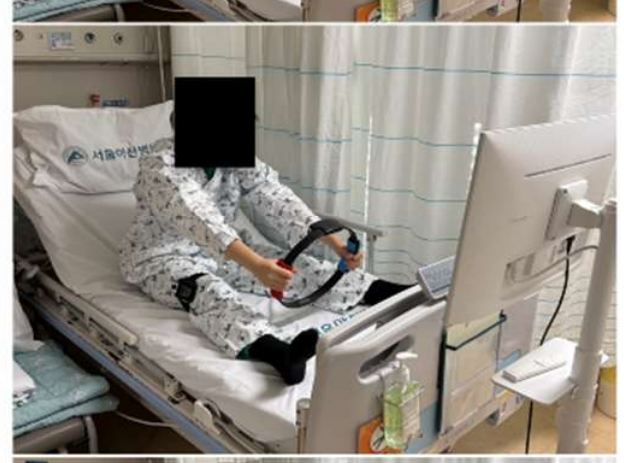	Trunk control and flexiblity
	Overhead side bend	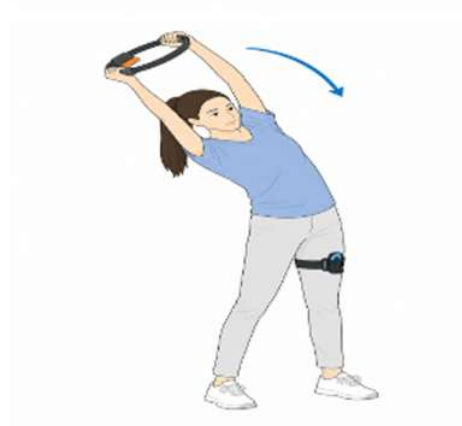	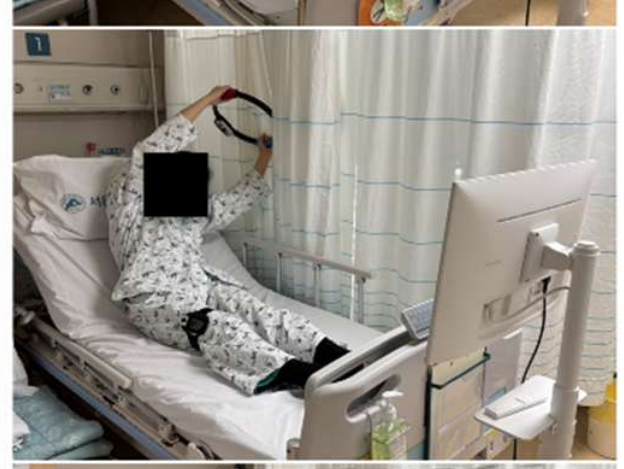	Trunk control and flexiblity
	Back press	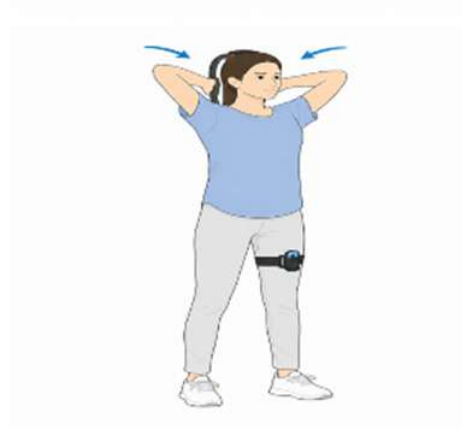	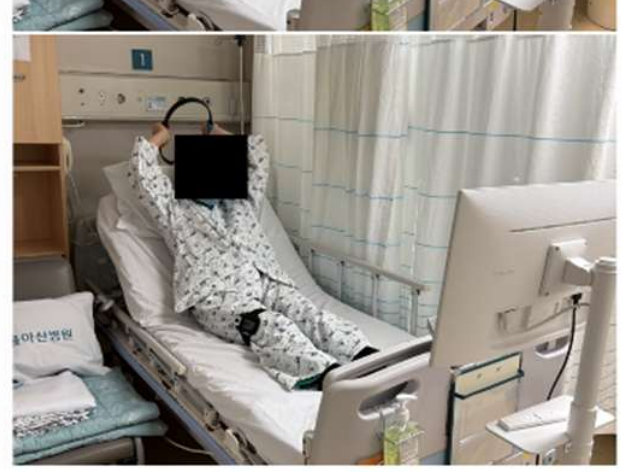	Trunk strengthening (posterior) with bilateral upper-extremity coordination

NFA: Nintendo Ring Fit Adventure.
