# Supplementary material for: Individually Prescribed In-Bed Exergaming Using Nintendo Ring Fit Adventure in Posttransplant Inpatients With Marked Functional Impairment After Intensive Care: Prospective Single-Arm Feasibility Study
Source: JMIR Serious Games. 2026 Oct 20;14:e95037. doi: 10.2196/95037 (PMC13684350; doi:10.2196/95037)
Supplement: Supplementary file 2 [file games-v14-e95037-s002.pdf]

Domain	Item number	Item	Response scale
Satisfaction and motivation	1	Exercising using this device is interesting.	1 = Strongly disagree; 2 = Disagree; 3 = Neutral; 4 = Agree; 5 = Strongly agree
	2	Overall, I am satisfied with the exercise program using this device.	
	3	I would like to continue exercising using this device.	
	4	Using this device increased my motivation to exercise.	
Convenience and suitability	5	Exercising using this device is convenient.	
	6	It was easy to understand how to perform the exercise program using this device.	
	7	I was able to perform the exercises without major difficulty (e.g., severe pain, dyspnea, or postural instability).	
Perceived effectiveness	8	This program helped me understand what kinds of exercises I should do in bed in the future.	

	9	This program helped increase my confidence in performing body movements.	
	10	My muscle strength improved after participating in this exercise program.	
	11	This program was beneficial to my overall health status.	
Perceived intensity	12	How would you rate the intensity of the provided exercise program?	1 = Very easy; 2 = Easy; 3 = Appropriate; 4 = Difficult; 5 = Very difficult
Free text	—	Please describe any positive aspects, inconveniences, or suggestions for improvement while using the program.	Open-ended response
