# Supplementary material for: Individually Prescribed In-Bed Exergaming Using Nintendo Ring Fit Adventure in Posttransplant Inpatients With Marked Functional Impairment After Intensive Care: Prospective Single-Arm Feasibility Study
Source: JMIR Serious Games. 2026 Oct 20;14:e95037. doi: 10.2196/95037 (PMC13684350; doi:10.2196/95037)
Supplement: Supplementary file 3 [file games-v14-e95037-s003.pdf]

Participant number / Age (years)	Transplant type	APACHE IV score	MV duration (days)	ICU stay (days)	Baseline FAC	Baseline ICUMS	Baseline MRC-SS	NFA sessions completed	Reason for non-completion / Relationship to the intervention
P07/31	DDLT	112	9	29	0	5	30	6	Transferred to ICU due to ICH (ruptured mycotic aneurysm) / Not related
P08/55	LDLT	94	20	22	0	7	36	6	Transferred to a rehabilitation hospital / Not related
P10/66	LDLT	89	27	32	1	8	30	3	Transferred to a rehabilitation hospital / Not related
P15/66	LDLT	94	7	4	2	8	38	5	Discharged home / Not related
P19/69	LDLT	93	31	33	0	1	24	6	Transferred to a rehabilitation hospital / Not related
P21/39	LDLT	85	13	19	1	8	36	1	Clinical deterioration (bilateral pelvic pain following a minor blunt trauma against a bed rail) / Not related

LDLT: living donor liver transplantation; DDLT: deceased donor liver transplantation; APACHE: Acute Physiology and Chronic Health Evaluation; MV: mechanical ventilation; ICU: intensive care unit; FAC: Functional Ambulation Category; ICUMS: Intensive Care Unit Mobility Scale; MRC-SS: Medical Research Council Sum Score; NFA: Nintendo Ring Fit Adventure; ICH: intracranial hemorrhage.
