# Supplementary material for: Individually Prescribed In-Bed Exergaming Using Nintendo Ring Fit Adventure in Posttransplant Inpatients With Marked Functional Impairment After Intensive Care: Prospective Single-Arm Feasibility Study
Source: JMIR Serious Games. 2026 Oct 20;14:e95037. doi: 10.2196/95037 (PMC13684350; doi:10.2196/95037)
Supplement: Supplementary file 4 [file games-v14-e95037-s004.pdf]

Measures category	Variable	χ^2	Overall *p* value		B-M change adjusted *p* value	M-F change adjusted *p* value	B-F change adjusted *p* value
Muscle strength and functional measures	MRC-SS	18.33	<.001***	Post hoc analysis	.02*	.09	.004**
	ICUMS	29.10	<.001***		.009**	.003**	<.001***
	FAC	24.50	<.001***		.03*	.004**	.001**
	Graded SLR	16.71	<.001***		.03*	.25	.01*
	Bridge test	15.20	<.001***		.07	.19	.01*
	HGS (kg)	19.00	<.001***		.07	.004**	.003**
	KES[1] (kg)	6.05	.049*		.06	.64	.06
HR-QOL (EQ-5D-5L)	EQ-5D index	3.89	.14	Post hoc analysis	-	-	-
	Mobility	14.12	<.001***		.92	.009**	.03*
	Self-care	4.19	.12		-	-	-
	Usual activities	1.64	.44		-	-	-
	Pain / Discomfort	0.57	.75		-	-	-
	Anxiety / Depression	4.53	.10		-	-	-
	EQ-VAS	10.93	.004**		.14	.18	.009**

HR-QOL: health-related quality of life; MRC-SS: Medical Research Council Sum Score; ICUMS: Intensive Care Unit Mobility Scale; FAC: Functional Ambulation Category; SLR: straight leg raise; HGS: handgrip strength; KES: knee extensor strength; EQ-VAS: EuroQol visual analog scale; B-M: baseline to midpoint; M-F: midpoint to final; B-F: baseline to final.

[1]KES was analyzed in participants with complete data at all assessment time points (n=12).
