# Supplementary material for: Individually Prescribed In-Bed Exergaming Using Nintendo Ring Fit Adventure in Posttransplant Inpatients With Marked Functional Impairment After Intensive Care: Prospective Single-Arm Feasibility Study
Source: JMIR Serious Games. 2026 Oct 20;14:e95037. doi: 10.2196/95037 (PMC13684350; doi:10.2196/95037)
Supplement: Supplementary file 5 [file games-v14-e95037-s005.pdf]

Targeting body part	Exercise type	Prescribed items	Completed items	Completion rate(%, 95% CI)
Total		2,161	2,072	95.9 (93.4–97.9)
Upper extremity		598 (27.7)	581 (28.0)	97.2 (94.5–99.2)
	Bow pull	223 (10.3)	221 (10.7)	99.1 (97.7–100.0)
	Overhead arm spin	198 (9.2)	188 (9.1)	94.9 (90.7–98.4)
	Overhead press	177 (8.2)	172 (8.3)	97.2 (93.6–99.5)
Lower extremity		649 (30.0)	628 (30.3)	96.8 (94.9–98.4)
	Thigh press	223 (10.3)	218 (10.5)	97.8 (95.5–99.6)
	Knee to chest	218 (10.1)	216 (10.4)	99.1 (97.7–100.0)
	Hip lift	208 (9.6)	194 (9.4)	93.3 (89.2–96.7)
Trunk		914 (42.3)	863 (41.7)	94.4 (90.4–97.6)
	Front press	211 (9.8)	203 (9.8)	96.2 (93.0–98.8)

Russian twist	203 (9.4)	187 (9.0)	92.1 (83.2–98.7)
Seated forward press	203 (9.4)	199 (9.6)	98.0 (95.6–100.0)
Seated ring raise	200 (9.3)	180 (8.7)	90.0 (83.7–95.3)
Overhead bend	46 (2.1)	45 (2.2)	97.8 (90.9–100.0)
Overhead side bend	41 (1.9)	40 (1.9)	97.6 (90.9–100.0)
Back press	10 (0.5)	9 (0.4)	90.0 (0.0–100.0)
