# Supplementary material for: Individually Prescribed In-Bed Exergaming Using Nintendo Ring Fit Adventure in Posttransplant Inpatients With Marked Functional Impairment After Intensive Care: Prospective Single-Arm Feasibility Study
Source: JMIR Serious Games. 2026 Oct 20;14:e95037. doi: 10.2196/95037 (PMC13684350; doi:10.2196/95037)
Supplement: Supplementary file 6 [file games-v14-e95037-s007.pdf]

No.	TIDieR item	Brief study-specific reporting	Where located
1	BRIEF NAME	Individually prescribed in-bed exergaming using NFA	Title and Abstract, pp 1–2
2	WHY	The program provided an engaging, adaptable form of bedside exercise for post-transplant inpatients whose activity after intensive care was limited by clinical and environmental barriers.	Introduction, p 2
3	WHAT: MATERIALS	Nintendo Switch console, Ring-Con, leg strap, NFA software, and height-adjustable bedside monitor; the exercise library and bedside adaptations are illustrated separately.	Methods, pp 3–5; Figure 1; Multimedia Appendix 1
4	WHAT: PROCEDURES	The therapist set up and calibrated the equipment, selected exercise items for intervention, supervised each session and provided assistance if needed, and applied prespecified safety-monitoring and stopping procedures.	Methods, pp 3–5; Figure 1; Multimedia Appendix 1
5	WHO PROVIDED	A certified physical therapist delivered all sessions. A physician confirmed readiness before each session; a physician or advanced practice nurse was consulted by therapist as needed.	Methods, p 5
6	HOW	Sessions were delivered individually and face-to-face under direct therapist supervision with the monitor and gaming equipment positioned for in-bed use.	Methods, p 5; Figure 1
7	WHERE	The intervention was delivered at the bedside on the general ward.	Methods, pp 3–5; Figure 1
8	WHEN AND HOW MUCH	Up to 10 once-daily sessions within a 30-minute treatment window; up to 10 selected items per session, typically performed as 1 set of 8-14 repetitions per exercise item. No prespecified quantitative intensity target was used.	Methods, p 5
9	TAILORING	Exercise selection, number of items, repetitions, body position, and manual assistance were individualized according to daily clinical status, functional capacity, and exercise response.	Methods, p 5; Figure 1; Multimedia Appendix 1
10	MODIFICATION S	Not applicable. The intervention protocol was not changed during the study.	Not applicable
11	HOW WELL: PLANNED	Intervention fidelity was assessed by the treating physical therapist using standardized session logs and prespecified session- and exercise-item–level definitions for prescribed, attempted, completed, and interrupted items. Ring-Con calibration was standardized before each session.	Methods, pp 5–6
12	HOW WELL: ACTUAL	Of 260 protocol-planned sessions, 227 were delivered and 223 were completed. Of 2161 prescribed exercise items, 2103 were attempted and 2072 were completed. Reasons for participant discontinuation, session interruption, and exercise-item interruption are reported.	Results, pp 7–8 and 12–13; Figure 2; Table 3; Multimedia Appendices 3 and 5
