# Supplementary material for: Individually Prescribed In-Bed Exergaming Using Nintendo Ring Fit Adventure in Posttransplant Inpatients With Marked Functional Impairment After Intensive Care: Prospective Single-Arm Feasibility Study
Source: JMIR Serious Games. 2026 Oct 20;14:e95037. doi: 10.2196/95037 (PMC13684350; doi:10.2196/95037)
Supplement: Supplementary file 7 [file games-v14-e95037-s006.docx]

Participant-level frequency distributions of prescribed and completed exercise items across 10 intervention sessions.
Data were obtained from 20 participants who completed all 10 intervention sessions (n=20 per session). Each cell indicates the number of participants with the corresponding exercise item count; darker colors indicate a greater number of participants. Blank cells indicate that no participant had the corresponding count. (A) Number of prescribed exercise items per participant at each session. (B) Number of completed exercise items per participant at each session.
